# Hybrid and Vaccine-Induced Immunity Against SARS-CoV-2 in a Cohort of Hospitalized Patients from the Metropolitan Aburrá Valley, Colombia

**DOI:** 10.3390/vaccines14050394

**Published:** 2026-04-28

**Authors:** Olga H. Hernández-Ortiz, Andrés F. Naranjo, Juan J. Vélez-Cadavid, Gisela De La Rosa, Bladimir A. Gil, A. Melissa Moreno, Laura S. Perez-Restrepo, Jaime Usuga, Manuela Aristizabal-Valencia, Francisco Molina-Saldarriaga, Jorge E. Sará-Ochoa, Natalia Betancourt-Rodriguez, Fabian Jaimes, Jorge E. Osorio, Juan Pablo Hernández-Ortiz

**Affiliations:** 1 One Health Genomic Laboratory, Universidad Nacional de Colombia, Medellín, Calle 75 N°79 A-51, Bloque M15, Medellín 050034, Colombia; anrobledom@unal.edu.co (A.M.M.); lab_cwohc@unal.edu.co (L.S.P.-R.); jausugar@unal.edu.co (J.U.); maaristizabal@unal.edu.co (M.A.-V.); jphernandezo@wisc.edu (J.P.H.-O.); 2 Clínica Medellín-Grupo Quirónsalud, Medellín 050024, Colombia; anaranjouci@gmail.com; 3 School of Medicine, Universidad de Antioquia, Medellín 050010, Colombia; fabian.jaimes@udea.edu.co; 4 Hospital General de Medellín, Medellín 050015, Colombia; jvelez@hgm.gov.co; 5 Hospital Pablo Tobón Uribe, Medellín 050015, Colombia; giseladlr@gmail.com; 6 Clínica las Américas-Auna, Medellín 050032, Colombia; bladigil@yahoo.com; 7 Clínica Universitaria Bolivariana, Medellín 050034, Colombia; francisco.molina@upb.edu.co; 8 Orlando Family Physicians, Miami, FL 34759, USA; jeso72@gmail.com; 9 Department of Pathobiological Sciences, School of Veterinary Medicine, University of Wisconsin-Madison, Madison, WI 53726, USA; nbetancourt@wisc.edu (N.B.-R.); jorge.osorio@wisc.edu (J.E.O.)

**Keywords:** COVID-19, SARS-CoV-2, mRNA vaccines, humoral immunity, cellular immunity, neutralizing antibodies, T cells, comorbidities, breakthrough infections

## Abstract

Background: Despite hybrid and vaccine-induced immunity, SARS-CoV-2 continues to cause disease. The characterization of humoral and cellular immune responses is essential for guiding prevention strategies and booster dose policies; Methods: A prospective cohort study was conducted with 131 hospitalized patients with confirmed COVID-19 in the Aburrá Metropolitan Valley, Colombia. Clinical and immunological data were evaluated on days 1–3, days 5–7, days 8–12, and 4–5 months after diagnosis. Humoral immunity was assessed by enzyme-linked immunosorbent assay (ELISA), chemiluminescent microparticle immunoassay (CMIA), and neutralization testing, and cellular immunity by CD4^+^/CD8 T-cell responses. Results: vaccinated patients had higher baseline levels of IgG and neutralizing antibody positivity than unvaccinated patients (ELISA 89.1% vs. 60.0%; CMIA 86.4% vs. 50.0%; neutralizing antibodies 88.2% vs. 65.0%), but cases of severe disease occurred in both groups. Adults aged ≥65 years had higher antibody positivity, but severe disease persisted. Mortality at 28 days was 7.6%, mainly among critically ill patients with comorbidities. Antibodies persisted at 4–5 months but were lower in those with severe acute disease. Those who received the booster dose showed stronger CD4^+^/CD8^+^ activation (notably against the Omicron variant) than unvaccinated/partially vaccinated patients. Conclusions: Vaccination improved humoral and cellular responses, but severe breakthrough infections still occurred, particularly in high-risk patients.

## 1. Introduction

Coronavirus disease 2019 (COVID-19), caused by severe acute respiratory syndrome coronavirus 2 (SARS-CoV-2), ranges from asymptomatic or mild infection to severe respiratory illness and systemic disease. A major determinant of this clinical heterogeneity is dysregulation of the host immune response, characterized by excessive innate immune activation, cytokine imbalance, and impaired adaptive immunity involving both T- and B-cell compartments. These alterations contributed substantially to the high morbidity and mortality observed during the early stages of the pandemic and remain closely linked to severe disease progression, multiorgan failure, death, and post-COVID syndrome, particularly among individuals with cardiovascular disease, diabetes, chronic respiratory disorders, and immunocompromising conditions, in whom altered immune function further increases the risk of poor outcomes [1,2,3].

In response to this global health emergency, countries worldwide implemented public health and social measures to reduce transmission and mitigate the burden of severe disease. At the same time, unprecedented international collaboration accelerated the development of multiple vaccines, making vaccination the cornerstone of the global response to COVID-19 [4]. In Colombia, five vaccine platforms were acquired through direct agreements with pharmaceutical companies and the COVAX mechanism (COVID-19 Vaccines Global Access), a World Health Organization-led initiative designed to support equitable vaccine distribution. This enabled the launch of the National COVID-19 Vaccination Plan on 17 February 2021 [5]. As in many other countries, the program initially prioritized healthcare workers, older adults, and individuals with comorbidities, while also adapting to changing vaccine availability and national operational needs.

The early phase of vaccination in Colombia relied mainly on the BNT162b2 messenger ribonucleic acid (mRNA) vaccine for healthcare workers and the inactivated CoronaVac vaccine for older adults, followed by broader deployment of viral vector vaccines such as ChAdOx1 nCoV-19 and Ad26.COV2.S, as well as the mRNA-1273 vaccine. Booster doses were first introduced for high-risk groups in September 2021 and were later extended to the general population, with coverage influenced by access, socioeconomic conditions, and vaccine acceptance [6]. As a result, Colombia developed a highly heterogeneous vaccination landscape shaped by the coexistence of multiple biologic platforms, variable schedules, and unequal uptake across population groups.

Overall, COVID-19 vaccination enhances neutralizing antibody production and promotes long-term cellular immune memory, thereby reinforcing protective immunity and reducing the risk of severe disease [7,8]. In line with these effects, vaccination has substantially reduced hospitalization and mortality rates and has played a central role in mitigating the burden of SARS-CoV-2 infection [9]. Nevertheless, the magnitude and durability of this protection vary across populations and remain under active investigation. Aging and comorbid conditions, two major determinants of poor clinical outcome, are frequently associated with early inflammation and a delayed, dysregulated adaptive immune response that may impair the development of effective T-cell immunity [1,2,3]. As a result, waning of vaccine-induced protection may be more pronounced in high-risk groups [10,11,12]. Consistent with this, follow-up studies have shown a gradual decline in protection over time, evidenced by recurrent outbreaks and peaks of infection despite completion of the primary vaccination schedule [13]. These observations have supported consideration of additional booster doses and heterologous vaccination strategies to strengthen and prolong immunity [14,15,16]. Consistent with this, breakthrough infections, defined as SARS-CoV-2 infections occurring despite full vaccination, have been reported more frequently among older adults and individuals with comorbidities [11,17,18,19], likely reflecting both baseline immune dysfunction and accelerated waning of vaccine-induced immunity. Importantly, the course of the pandemic was not uniform across countries. Variations in viral circulation, vaccine availability, and immunization strategies shaped distinct epidemiologic and immunologic landscapes, underscoring the need for context-specific studies of post-infection and post-vaccination immunity.

Colombia represents one such particularly relevant context. Sustained co-circulation of multiple SARS-CoV-2 lineages, including the regionally significant Mu variant, preceded and overlapped with the emergence of Delta and Omicron. This distinctive viral landscape, combined with the concurrent deployment of multiple vaccine platforms, generated a heterogeneous and dynamic immune environment, making Colombia an especially informative setting for the study of long-term immunity in low- and middle-income countries [20,21,22]. Within this setting, the convergence of variant co-circulation, diverse vaccine exposure, and variable timing of infection and vaccination created a real-world model of complex hybrid immunity that extends beyond classical definitions.

The objectives of this study were to characterize the status of natural and vaccine-induced immunity during acute SARS-CoV-2 infection and to evaluate the immune profile of participants who underwent follow-up sampling 4–5 months after recovery from acute COVID-19 in the Metropolitan Area of the Aburrá Valley, Colombia. We sought to describe the maintenance of immunity over time according to vaccination status, with particular emphasis on booster vaccination, to characterize the immunological patterns observed across vaccine platforms during follow-up, and to document genomic surveillance findings relevant to potential immune escape.

## 2. Materials and Methods

### 2.1. Study Design

Prospective cohort study with longitudinal data collection.

### 2.2. Settings

The study was conducted between October 2021 and February 2023 at eight tertiary care centers in Medellín, Colombia, including Hospital General de Medellín (HGM), Clínica Las Américas-Auna (CLA), Hospital Pablo Tobón Uribe (HPTU), Hospital Universitario San Vicente Fundación (HUSVF), Clínica Alma Mater de Antioquia (CAM), Clínica Medellín-grupo Quirónsalud (CM), Clínica CES, and Clinica Universidad Bolivariana (CUB).

### 2.3. Participants

The study included patients aged 18 years and older with acute SARS-CoV-2 infection who were hospitalized in general wards or intermediate/intensive care units. Exclusion criteria comprised the presence of a do-not-resuscitate order and the lack of a contact phone number for follow-up.

Participants were followed up on day 28 post-infection through telephone interviews, during which a standardized form was completed to document their vital status. Follow-up continued until the sixth month.

The study protocol was approved by the ethics committee of each participating institution, and informed consent was obtained from all participants or their legal representatives prior to enrollment.

### 2.4. Descriptive Variables

#### 2.4.1. Vaccination Status

Vaccination status was assessed within the first 72 h of admission by measuring anti-SARS-CoV-2 antibodies, nucleocapsid antibodies, and neutralizing antibodies against the Wuhan, Mu, BA.1, and BA.2 variants. Among participants who consented to follow-up sampling, the same markers were reassessed 4–5 months after acute COVID-19 to evaluate immune status over time, with repeat neutralization testing performed in those initially negative for neutralizing antibodies.

#### 2.4.2. Cellular Immunity

CD4^+^ and CD8^+^ T-cell counts (cells/mL) were measured at three time points within the first 12 days of hospitalization and again at 4–5 months following COVID-19 diagnosis.

#### 2.4.3. Viral Variants

SARS-CoV-2 genomes obtained from patients during acute infection were sequenced and classified using three complementary systems: World Health Organization (WHO) Greek-letter variant nomenclature, Pango lineage assignment, and Nextclade clade classification [23,24,25].

#### 2.4.4. Clinical Classification of Acute SARS-CoV-2 Infection [26]

Moderate Disease: Clinical manifestations of pneumonia (fever, cough, dyspnea, and tachypnea) with oxygen saturation greater than 94% on room air.

Severe Disease: Pneumonia accompanied by a respiratory rate greater than 30 breaths per minute, evidence of respiratory distress or oxygen saturation less than 94% on room air, a PaO_2_/FiO_2_ ratio (PaFI) less than 300 mmHg, or pulmonary opacities involving more than 50% of the lung parenchyma within the first 24–48 h.

Critical Disease: Presence of septic shock, cardiac involvement, respiratory failure, and/or multiple organ dysfunction syndrome.

#### 2.4.5. Potential Confounders

Potential confounders included age, sex, and underlying medical conditions associated with an increased risk of severe COVID-19, as defined by the Centers for Disease Control and Prevention (CDC) [27]. Comorbidities were grouped as chronic lung disease (asthma or chronic obstructive pulmonary disease), neoplasia (solid tumors, leukemia, or lymphoma), weakened immune system status (including neoplasia, solid organ or hematopoietic stem cell transplantation, HIV infection, lupus, or rheumatoid arthritis), and other high-risk conditions (chronic lung disease, diabetes, obesity, chronic kidney disease, cerebrovascular disease, coronary artery disease, and hypertension). A composite medical condition variable was defined as the presence of weakened immune system status and/or other high-risk conditions.

### 2.5. Data and Sample Collection

Each participant completed a clinical-epidemiological survey to gather demographic data, medical history, vaccination details, symptom progression, past medical conditions, immunosuppressive history, and socioeconomic and cultural background. Additionally, a follow-up survey on acute infection was conducted, with participants contacted at three and six months post-infection to assess any emerging or persistent symptoms indicative of post-COVID-19 syndrome. Trained One Health personnel and participating physicians at each center administered the survey.

Vaccination records were obtained from participants’ immunization cards and cross-referenced with official databases, including PAIWEB (which tracks individual vaccine administration). Additionally, follow-up phone calls were made to collect any missing data.

### 2.6. Measurement of Immune Response

Peripheral blood samples were collected within 72 h of admission, again on days 4–7 and 8–12 after diagnosis, and at 4–5 months in participants who consented to long-term follow-up. Humoral immune responses were assessed using the SCoV-2 Detect™ IgG ELISA Kit (InBios International, Inc., Seattle, WA, USA), the SARS-CoV-2 IgG II assay (Abbott Laboratories, Abbott Park, IL, USA), the SARS-CoV-2 IgM Reagent assay (Abbott Abbott Laboratories, Abbott Park, IL, USA), a Luminex xMAP-based assay for quantitative detection of anti-nucleocapsid IgG using the anti-IgG nucleocapsid panel (Invitrogen, Thermo Fisher Scientific, Waltham, MA, USA), and the cPass SARS-CoV-2 Neutralization Antibody Detection Kit (GenScript USA Inc., Piscataway, NJ, USA). For the InBios ELISA, assay validity was established according to internal quality control criteria: mean positive control (CP) ≥ 0.85, mean negative control (CN) < 0.25, and cut-off control (CCO) within the range of 0.25 to 0.85. For the Abbott CMIA assays, results were interpreted according to the manufacturer’s cut-offs: IgM was considered negative if COV < 1.0 and reactive if ≥1.0, whereas IgG was considered negative if <50.0 AU/mL and reactive if ≥50.0 AU/mL. For the anti-nucleocapsid IgG assay, results were evaluated using mean fluorescence intensity (MFI), with healthy control samples included in each run to define cutoffs between negative and positive PCR samples. A relative index was calculated as the ratio between sample MFI and low-control MFI, and results were classified as negative (<1.0), indeterminate (1.0–1.3), or positive (>1.3). The low-control threshold had been previously established using a reference cohort of 160 PCR-negative and 39 PCR-positive SARS-CoV-2 samples. When a high-concentration control was available, standard curves were generated to estimate relative quantitative values (U/mL) using ProcartaPlex™ Analyst software, version 1.0, available on the Thermo Fisher Connect™ Platform (Thermo Fisher Scientific, Waltham, MA, USA). Neutralizing antibodies were assessed in serum using the cPass assay with variant-specific reagents for Wuhan, Mu, BA.1, and BA.2, and positivity was defined as ≥30% inhibition according to the manufacturer’s instructions. These criteria were applied uniformly across all samples and analyses. For participants with initially negative neutralizing antibody results, repeat quantitative testing was performed at follow-up.

Cellular immune responses were assessed by flow cytometry, with peripheral blood mononuclear cells (PBMCs) isolated at each time point, fixed, permeabilized, and stained with fluorochrome-conjugated monoclonal antibodies (Becton Dickinson, San Jose, CA, USA) from the TBMNK Backbone Panel. CD4^+^ and CD8^+^ T-cell counts (cells/mL). The gating strategy included exclusion of debris, lymphocyte selection, and doublet discrimination before analysis of the relevant cell populations. Potential artifacts were minimized through this standardized gating approach based on scatter properties and sequential population selection (Supplementary S1: Table S2 and Figure S1).

All samples were processed in a Biosafety Level 2+ (BSL-2+) research laboratory at the One Health Genomic Laboratory, Universidad Nacional de Colombia-Medellín campus, following standardized biosafety and analytical protocols (Supplementary S1: Methods).

### 2.7. SARS-CoV-2 Genomic Surveillance

Within 72 h of enrollment, nasal swab samples were collected for quantitative reverse transcription polymerase chain reaction (qRT-PCR) and genomic surveillance of acute infection. Viral ribonucleic acid (RNA) was extracted using the ZR Viral RNA Kit (Zymo Research, Irvine, CA, USA), and SARS-CoV-2 was detected with the Berlin-Charité protocol targeting the E and RNA-dependent RNA polymerase (RdRp) genes [28] (Supplementary S1: Table S1). Samples with a cycle threshold (Ct) value < 38 were considered positive, and those with Ct < 27 were selected for whole-genome sequencing using the nCoV-2019 v4.1 protocol [29]. Raw reads were processed with the Oxford Nanopore Technologies (ONT) pipeline, and consensus genomes were classified using Pango lineage assignment and Nextclade. Phylogenetic analysis was performed on approximately 5000 complete SARS-CoV-2 genomes retrieved from the Global Initiative on Sharing All Influenza Data (GISAID), following the method of Hadfield et al. [30] with Augur v24.1.0 [31]. Sequences were aligned to reference genome MN908947.3, trees were inferred with IQ-TREE v1.6.1 [32], temporally refined, and visualized in Auspice.

### 2.8. Bias

The principal investigator audited records to ensure data integrity, triggering re-evaluation for extreme or inconsistent values. Trained personnel conducted standardized surveys to minimize interviewer bias and maintain consistency. Participants were enrolled consecutively upon consent, with contact information recorded for follow-up. All laboratory analyses were performed in a certified facility under strict quality control protocols to ensure data accuracy and reliability.

### 2.9. Study Size

A descriptive analysis was conducted for the entire cohort. This study is part of the project “Immune Response Against SARS-CoV-2 and its Association with Mortality, Organ Dysfunction, and Post-COVID Syndrome: Characterization of Immunity and Genomic Surveillance of Viral Variants.” The cohort included hospitalized patients from the metropolitan area of the Aburrá Valley. Sample size calculations were performed (Supplementary S1: Sample size, Table S3).

### 2.10. Quantitative Variables

“Age” and “time since vaccination” were dichotomized based on literature thresholds: 65 years or older and ≥4 months since vaccination.

### 2.11. Statistical Methods

Descriptive statistics were used to summarize baseline characteristics and study variables. Continuous variables were reported as mean ± standard deviation or median (interquartile range [IQR], range), according to their distribution, and categorical variables as frequencies and percentages. Longitudinal changes in seropositivity between Measurement 1 and Measurement 4 were assessed using McNemar’s test among participants with valid paired samples, focusing on discordant pairs, including seroconversion and seroreversion events. The exact binomial version of McNemar’s test was used when fewer than 25 discordant pairs were observed; otherwise, the chi-square approximation with Yates’ continuity correction was applied. Cross-sectional analyses at Measurement 1 compared antibody positivity according to vaccination status and age group (<65 vs. ≥65 years), with additional stratification by disease severity (critical vs. non-critical); Fisher’s exact test was used when subgroup sizes were small. To assess longitudinal changes in CD4^+^ and CD8^+^ T-cell subsets across the four predefined time points, linear mixed-effects models were fitted to log10(x + 1)-transformed absolute counts, with time point, primary vaccine platform, booster strategy, and their interactions included as fixed effects, and participant identifier included as a random effect. Post hoc chronological comparisons were performed using Welch’s *t*-test on the transformed data. All tests were two-sided, and *p*-values < 0.05 were considered statistically significant. These analyses were considered exploratory and were interpreted with appropriate caution. (Supplementary S1: Statistical Methods)

Missing data was imputed using the Predictive Mean Matching (PMM) method in the MICE package (R software, version 4.4.1; R Foundation for Statistical Computing, Vienna, Austria), considering age, sex, COVID-19 severity, comorbidities, vaccination, and booster status [22]. (Supplementary S1: Missing values). Analyses were performed using Stata 17 (StataCorp LLC, College Station, TX, USA) and R/RStudio (version 4.4.1, R Core Team).

## 3. Results

### 3.1. General Characteristics

**Figure 1:**
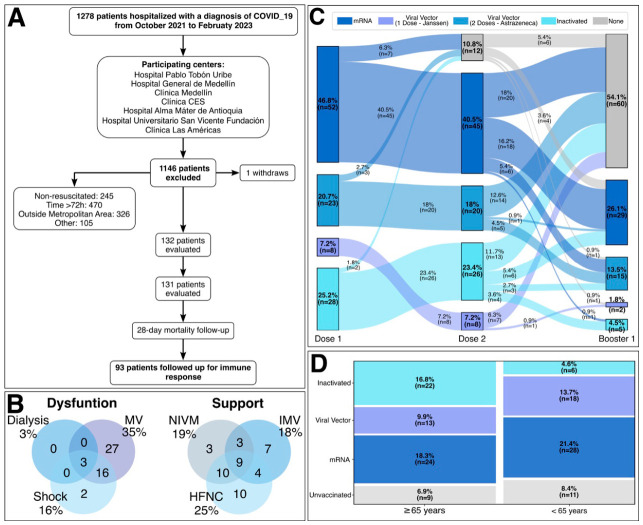
Study design, clinical characteristics, and vaccination profile of the cohort. (**A**) Flowchart illustrating patient enrollment and follow-up of hospitalized individuals with laboratory-confirmed COVID-19 included in the prospective cohort. (**B**) Venn diagram showing the overlap between organ dysfunction and the need for ventilatory support among hospitalized patients. (**C**) Proportion of individuals in the cohort who met the requirements for a complete primary COVID-19 vaccination schedule, stratified by vaccine type (n = 111). (**D**) Distribution of vaccine platforms administered as part of the initial vaccination schedule in the study population, stratified by age group (<65 years and ≥65 years) (n = 131).

During the study period, 1278 patients were hospitalized with SARS-CoV-2 infection, and 131 were included in the cohort between October 2021 and February 2023. Of these, 63 were recruited at HPTU, 20 at CM, 18 at CLA, 13 at HUSVF, 8 at CAM, 8 at CES, and 2 at HGM. Complete follow-up was achieved at 28 days, including assessment of vaccination status and immune response. These evaluations were repeated at 4 to 5 months after COVID-19 diagnosis in a subset of 93 patients (see Figure 1A).

The median age was 65 years (interquartile range (IQR): 56 to 75); 53.4% were female and 65% were older than 50 years. Most patients (84%) had at least one high-risk comorbidity, mainly hypertension (55%), diabetes (24%), and chronic respiratory disease (24%). Overall, 84% were vaccinated against SARS-CoV-2, and 39% had received a booster dose. Cough and dyspnea were the most frequent symptoms. Disease severity was moderate in 22.1%, severe in 41.2%, and critical in 36.6% of patients. Among patients with critical illness, organ dysfunction was observed in all cases. Overall, 95.8% (21/22) required ventilatory support due to respiratory failure, 43.7% (7/16) developed shock necessitating vasopressor therapy, and 6.2% (3/48) required renal replacement therapy. The most frequent combination of advanced supportive measures was ventilatory support plus vasopressors, administered to 16 patients (33%). Respiratory support modalities included invasive mechanical ventilation (IMV), non-invasive ventilation (NIV), and high-flow nasal cannula (HFNC), with non-invasive or hybrid strategies being the most employed. (see Figure 1B). The 28-day mortality was 7.6% (10/131), higher in critically ill than non-critical patients (18.8% vs. 1.2%). Mortality was 19.5% among ventilated patients, rising to 33% with shock or renal replacement therapy and to 37.5% with combined mechanical ventilation and vasopressors. Six of nine patients (66.6%) requiring all three respiratory support modalities died (Table S4).

### 3.2. Vaccination

Among the 111 vaccinated patients enrolled in the study, at baseline, missing data included one nucleocapsid antibody measurement, two spike antibody results obtained by CMIA, and ten neutralizing antibody results, representing 2.48% of the total data for these variables. Missing values were imputed using the previously described method (Supplementary S1: Missing values).

Overall, 85% were vaccinated against SARS-CoV-2, and 39% had received a booster dose. At study entry, 20 patients (15%) were unvaccinated. Regarding vaccination status, 46.8% (52/111) received an mRNA vaccine as the first dose, 20.7% (23/111) AstraZeneca, 7.2% (8/111) Janssen, and 25.2% (28/111) an inactivated vaccine. Among those initiating a two-dose regimen, the primary series was incomplete in 13.4% (7/52) of mRNA recipients, 13.0% (3/23) of AstraZeneca recipients, and 7.1% (2/28) of those receiving inactivated vaccines. Overall, 10.8% had an incomplete primary vaccination schedule.

A total of 51 participants (46%) received a booster dose; mRNA vaccines were the predominant booster platform (*n* = 29, 57%). A homologous booster was defined as receipt of the same vaccine platform used in the primary vaccination series, whereas a heterologous booster was defined as receipt of a different vaccine platform. Among those primed with an mRNA vaccine *(n* = 29), 18 received a homologous mRNA booster, including four with an incomplete primary series, whereas seven received a heterologous booster, including six viral vector and one inactivated vaccine. Among AstraZeneca-primed participants (*n* = 6), five received a homologous booster and one received a heterologous mRNA booster; additionally, one participant with an incomplete AstraZeneca primary series received a Janssen booster. Among those primed with an inactivated vaccine (*n* = 14), four received a homologous booster and ten received a heterologous booster. Of the latter, four received a viral vector booster and six received an mRNA booster (Figure 1C). The median time from the last vaccine dose to study enrollment was 304 days (IQR, 202–496). Among boosted participants, the median interval between booster administration and enrollment was 200 days (IQR, 136–326).

Of the 68 participants aged ≥ 65 years (51.9%), non–mRNA vaccines predominated (*n* = 35; 59.3%) compared with mRNA vaccines (*n* = 24; 40.7%) (Figure 1D).

### 3.3. Serostatus Dynamics Using Nucleocapsid IgG, ELISA IgG, and CMIA Assays

**Figure 2:**
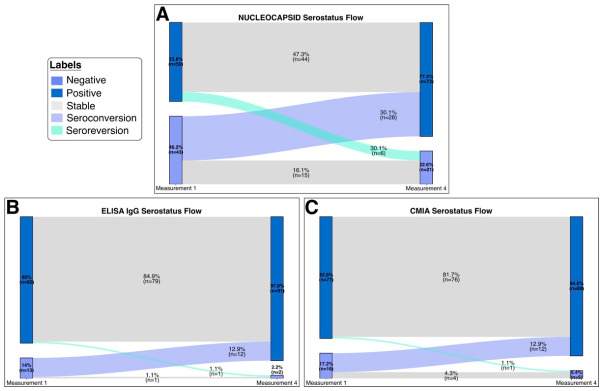
Longitudinal serological responses to SARS-CoV-2 infection. (**A**) Flow diagram showing the evolution of nucleocapsid antibody serostatus among patients across study time points (*n* = 93). (**B**) Flow diagram illustrating changes in anti–SARS-CoV-2 antibody serostatus measured by ELISA throughout follow-up (*n* = 93). (**C**) Flow diagram depicting the longitudinal serostatus of anti–SARS-CoV-2 antibodies measured by chemiluminescent microparticle immunoassay (CMIA) during the study period (*n* = 93).

Immune status was assessed in 93 patients 4 to 5 months after diagnosis and compared with baseline measurements obtained at enrollment; for nucleocapsid IgG, baseline seropositivity was 53.8%, increasing to 77.4% at follow-up, while seronegativity declined from 46.2% to 22.6%. Nucleocapsid positivity changed significantly between measurements (*p* < 0.001). Overall, 30.1% of participants seroconverted, and 6.5% lost detectable nucleocapsid antibodies. (Figure 2A). In contrast, spike-specific IgG measured by ELISA showed high baseline seropositivity at 86%, increasing to 97.8% at Measurement 4. This change in ELISA positivity between measurements was statistically significant (*p* = 0.0034). Seronegativity declined from 14% to 2.2%, with 12.9% of participants seroconverting and 1.1% becoming seronegative over time. (Figure 2B). Similarly, CMIA showed baseline seropositivity of 82.8%, increasing to 94.6% at follow-up; this change was statistically significant (*p* = 0.0034). Seronegativity declined from 17.2% to 5.4%, with 12.9% of participants seroconverting and 1.1% losing detectable antibodies (Figure 2C).

Variant-specific analysis of neutralizing antibody (Wuhan, Mu, BA.1, BA.2) revealed that, at baseline, positivity was highest for the Wuhan variant (*n* = 99; 75.6%) and BA.2 (*n* = 94; 71.8%). At 4–5 months, positivity rates were highest for BA.1 and BA.2 (Figure S2).

### 3.4. Anti-SARS-CoV-2 Antibodies by Vaccination Status and COVID-19 Severity

Among the 20 unvaccinated patients at enrollment, immunity assessment showed positive IgG anti-SARS-CoV-2 results in 10 patients (50.0%) by CMIA and in 12 patients (60.0%) by ELISA, compared with 86.4% and 89.1%, respectively, among vaccinated patients. Neutralizing antibodies were detected in 65.0% of unvaccinated patients compared to 88.2% of vaccinated patients.

**Figure 3:**
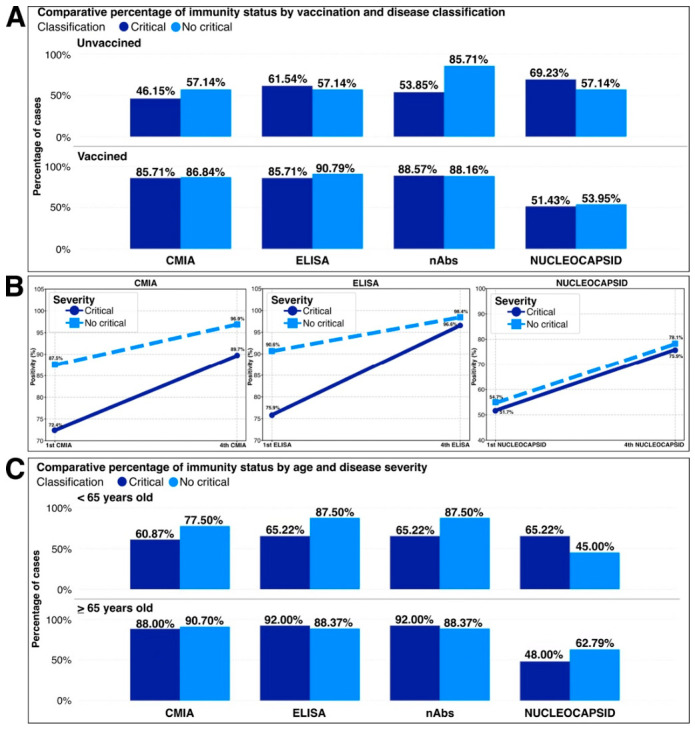
Immune response profiles according to vaccination status, disease severity, and age. (**A**) Distribution of humoral immunity status according to vaccination status and clinical disease severity. (**B**) Anti–SARS-CoV-2 antibody responses measured by different immunological assays, stratified by disease severity. (**C**) Immunity status according to age group (<65 years and ≥65 years) and disease severity.

At baseline, 48 patients presented with critical COVID-19; of these, 36 (73.5%) were vaccinated. Among those with non-critical disease, 75 patients (91.5%) were vaccinated. Severe COVID-19 occurred despite prior vaccination and positive immunologic test results. Among patients with critical COVID-19, CMIA positivity (*p* = 0.0090) and neutralizing antibody positivity (*p* = 0.0120) were higher in vaccinated patients, whereas ELISA positivity was numerically higher but did not differ significantly by vaccination status (*p* = 0.1075). Among those with non-critical disease, ELISA positivity (*p* = 0.0348) and neutralizing antibody positivity (*p* = 0.0296) also differed by vaccination status. (Figure 3A).

At 4–5 months follow-up, positivity of immunological assays remained high; however, positivity rates were lower among patients who had developed critical manifestations during acute infection (Figure 3B).

### 3.5. Immunity by Age Group

Because age ≥ 65 years is a known risk factor for COVID-19, this population was prioritized for vaccination in Colombia. At study entry, immunity was higher among patients aged ≥ 65 years compared with those < 65 years. Among patients ≥ 65 years with critical COVID-19, ELISA positivity (*p* = 0.0335) and CMIA positivity (*p* = 0.045) were higher than in those < 65 years, whereas neutralizing antibody positivity was also numerically higher but did not differ significantly by age (*p* = 0.353). Among patients with non-critical disease, CMIA and nucleocapsid positivity were also numerically higher in older adults, but these differences were not statistically significant. When comparing clinical severity by age, despite higher seropositivity among patients ≥ 65 years, this group continued to present with critical disease (Figure 3C).

### 3.6. Dynamics of CD4^+^ and CD8^+^ T-Cell Responses

This analysis was performed in the subgroup of patients with complete measurements at the predefined time points and included individuals who had received only the primary vaccination series, those who had received a homologous or heterologous booster, and unvaccinated participants.

### 3.7. Primary Vaccination Series (PVS) Stratified by Vaccine Platform or Unvaccinated

**Figure 4:**
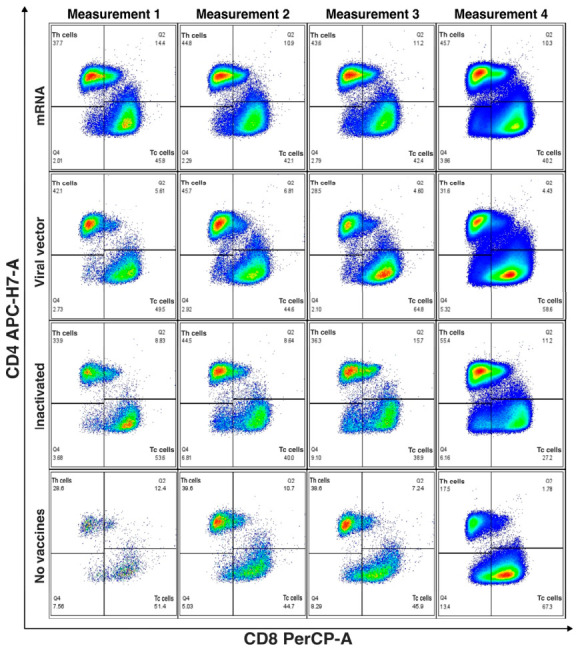
Longitudinal distribution of CD4^+^ and CD8^+^ T-cell subsets according to primary vaccination status. Representative bivariate flow cytometry dot plots show CD4 vs. CD8 expression within the lymphocyte gate across four predefined time points (Measurements 1–4). Quadrants identify CD4^+^ T cells, CD8^+^ T cells, CD4^+^CD8^+^ double-positive, and double-negative populations. **Top row**: mRNA-primed individuals. **Second row**: viral vector–primed individuals. **Third row**: inactivated vaccine–primed individuals. **Bottom row**: unvaccinated individuals.

At study entry, 60 patients had received only PVS, and 20 were unvaccinated (Figure 4). Across vaccine platforms, vaccinated participants generally showed preserved CD4^+^ T-cell proportions during the first 72 h, followed by expansion of CD8^+^ T cells at days 5–7 and a more balanced CD4^+^/CD8^+^ distribution by days 8–12 and at 4–5 months. This pattern was most consistent in the mRNA and viral vector groups, whereas participants vaccinated with inactivated platforms showed similar but more variable profiles over time. In contrast, unvaccinated participants showed lower CD4^+^ T-cell proportions at baseline and greater variability in both CD4^+^ and CD8^+^ distributions throughout follow-up. Overall, vaccinated individuals tended to maintain more preserved and balanced CD4^+^/CD8^+^ profiles, whereas unvaccinated participants exhibited less consistent patterns.

Compared with no vaccination, mRNA vaccination was associated with significantly higher and more sustained CD8^+^ T-cell levels from Measurement 1 to Measurement 4 (*p* = 0.045). No significant longitudinal differences were identified for the viral vector or inactivated vaccine platforms. Furthermore, a statistically significant increase in CD4^+^ T-cell levels from Measurement 1 to Measurement 3 was observed exclusively among participants who received an mRNA-based primary vaccination regimen (*p* = 0.0266).

### 3.8. Primary Vaccination Series (PNV) and Booster Strategy Stratified by Vaccine Platform

**Figure 5:**
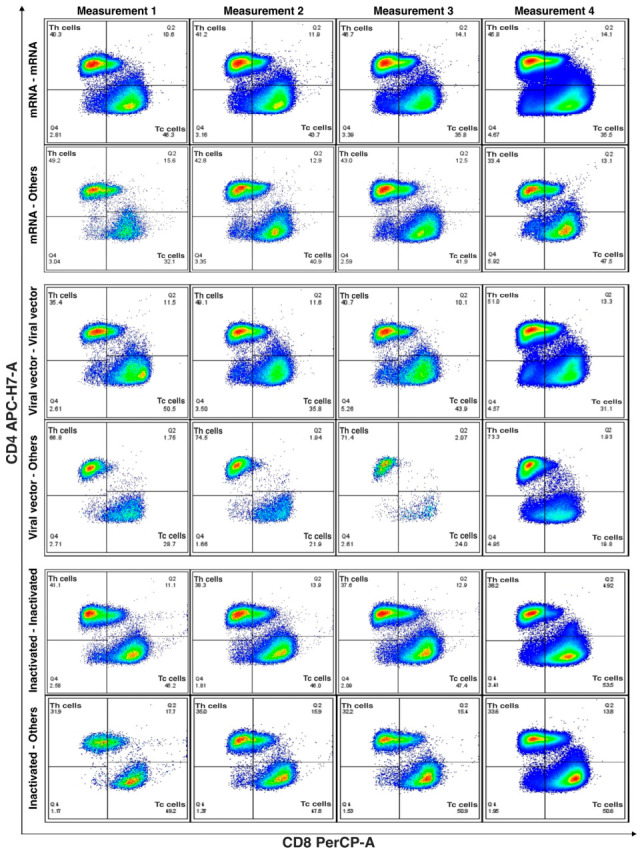
Longitudinal distribution of CD4^+^ and CD8^+^ T-cell subsets according to vaccination status and booster strategy. Representative bivariate flow cytometry dot plots show CD4 vs. CD8 expression within the lymphocyte gate across four predefined time points (Measurements 1–4). Quadrants identify CD4^+^ T cells, CD8^+^ T cells, CD4^+^CD8^+^ double-positive, and double-negative populations. **First row**: mRNA-primed, homologous booster. **Second row**: mRNA-primed, heterologous booster. **Third row**: viral vector–primed, homologous booster. **Fourth row**: viral vector–primed, heterologous booster. **Fifth row**: inactivated vaccine–primed, homologous booster. **Sixth row**: inactivated vaccine–primed, heterologous booster. Across panels, plots illustrate temporal shifts in CD4^+^ and CD8^+^ T-cell distributions from the acute phase through long-term follow-up.

Among the 51 participants who received a booster dose, CD4^+^ and CD8^+^ T-cell distributions were evaluated at the predefined time points according to the biologic platform used in the primary series and the type of booster received, homologous or heterologous (Figure 5). Overall, boosted participants maintained detectable CD4^+^ and CD8^+^ T-cell populations throughout follow-up. The most preserved and balanced profiles were generally observed in participants primed with mRNA platforms, whereas those primed with viral vector or inactivated vaccines showed comparable persistence of both T-cell compartments but with more heterogeneous distributions over time. Across platforms, both homologous and heterologous booster regimens were associated with maintained CD4^+^ and CD8^+^ T-cell detection at all evaluated time points.

Among vaccinated participants, homologous booster vaccination was associated with significantly higher CD8^+^ T-cell levels at Measurement 4 compared with no booster. In the CD4^+^ compartment, the longitudinal model showed a trend toward statistical significance at Measurement 4 among participants who received a homologous booster (*p* = 0.059).

### 3.9. SARS-CoV-2 Variants in CD4^+^ and CD8^+^ Subsets

**Figure 6:**
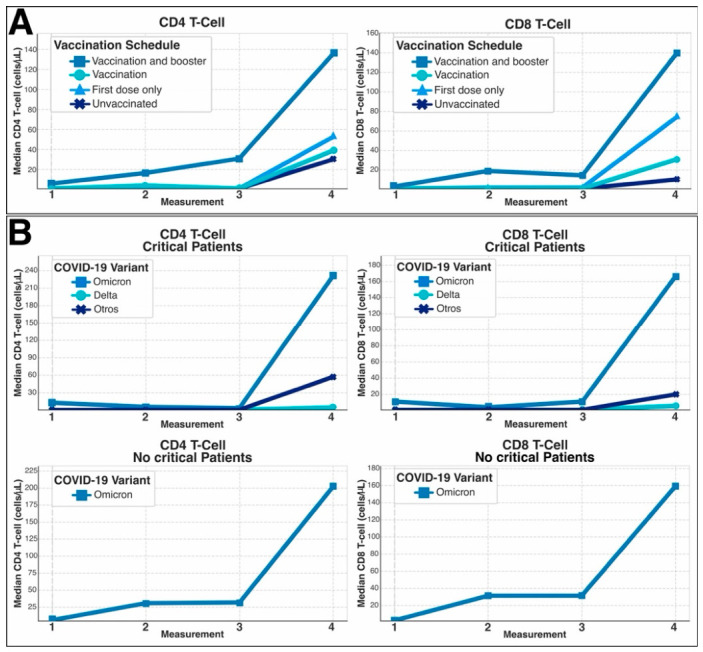
T-cell dynamics according to vaccination status, disease severity, and SARS-CoV-2 variant. (**A**) CD4^+^ and CD8^+^ T-cell counts stratified by vaccination schedule. (**B**) Median CD4^+^ and CD8^+^ T-cell counts stratified by clinical severity (critical vs. non-critical patients) and infecting SARS-CoV-2 variant (Omicron, Delta, or other variants).

Longitudinal median CD4^+^ and CD8^+^ T-cell counts were evaluated across four predefined time points (Measurements 1 to 4) according to vaccination schedule. During the acute phase (Measurements 1 to 3), median CD4^+^ and CD8^+^ counts remained low across all groups. At Measurement 4 (4 to 5 months post-diagnosis), an increase in both CD4^+^ and CD8^+^ T-cell counts was observed in all vaccination categories. The highest median counts at follow-up were observed among individuals who had received vaccination plus booster, whereas lower median values were observed in partially vaccinated and unvaccinated individuals (Figure 6A).

Among patients with critical disease (upper panel), a recovery occurred later, with an abrupt increase at the fourth measurement in those infected with the Omicron variant, both in CD4^+^ and CD8^+^ subsets. In contrast, Delta and other variants showed more attenuated responses, with persistently low counts. In patients with non-critical disease (down panel), Omicron infection was also associated with a progressive rise in T-cell counts, particularly by the fourth measurement, reaching values comparable to or higher than those observed in critical cases. This trend was especially pronounced in CD4^+^ and CD8^+^ subsets (Figure 6B).

### 3.10. SARS-CoV-2 Genomic Surveillance

**Figure 7:**
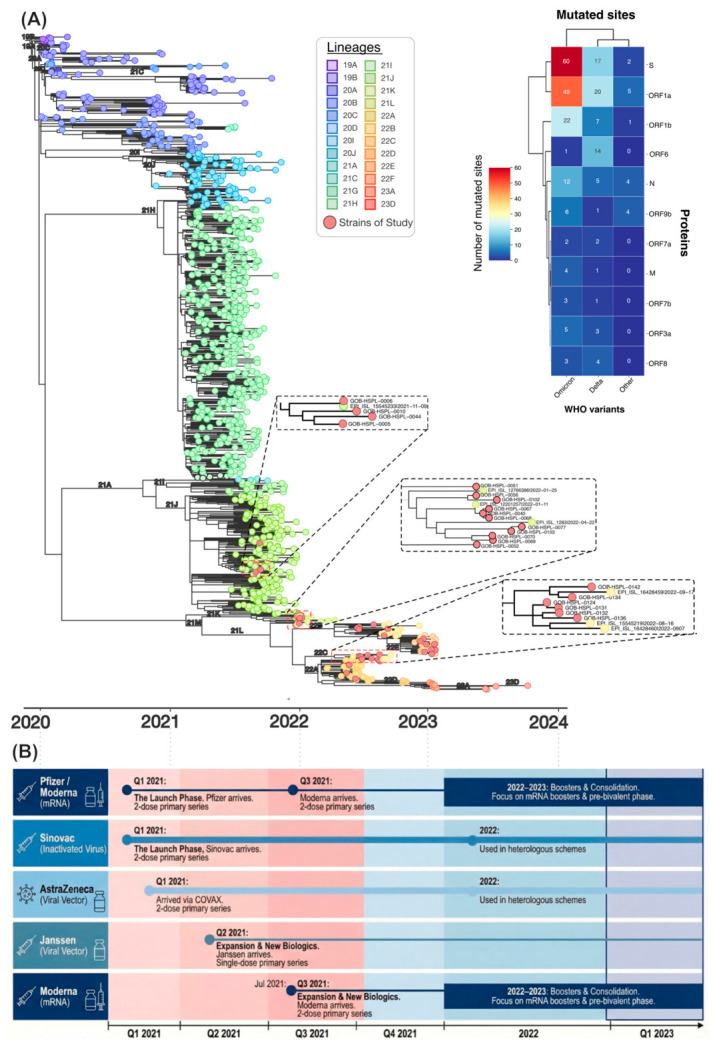
Phylogenetic structure, mutational landscape of SARS-CoV-2 genomes in Colombia, and national vaccination timeline (2020–2023). (**A**) Time-resolved phylogenetic tree of approximately 5000 complete SARS-CoV-2 genomes from Colombia (2020–2023) obtained from the GISAID database. Phylogenetic reconstruction followed the protocol of Hadfield et al., (2021) [30] using the Nextstrain Augur pipeline (v24.1.0). Sequences were aligned to the Wuhan-Hu-1 reference genome (MN908947.3) using augur align. Maximum-likelihood phylogeny was inferred with IQ-TREE v1.6.1 and temporally refined using augur refine. Clades were assigned according to Nextstrain nomenclature using augur clades, based on nucleotide and amino-acid substitutions inferred with augur ancestral and augur translate. The final tree was visualized in Auspice. Genomes generated in this study are highlighted in red and indicated by dashed boxes. The accompanying heatmap shows the distribution of mutated sites by viral gene across major WHO-designated variants (Omicron, Delta, and Gamma). Multiple sequence alignment was performed with MAFFT, and mutations were identified relative to the Wuhan-Hu-1 reference genome (MN908947.3). Colors represent the number of mutated sites per gene (warmer colors indicate higher mutation counts). Hierarchical clustering highlights similarities in mutational patterns among variants. (**B**) Timeline of Colombia’s COVID-19 vaccination rollout (Q1 2021–Q1 2023). Initial deployment prioritized BNT162b2 (Pfizer–BioNTech, mRNA) for healthcare workers and CoronaVac (Sinovac, inactivated) for older adults, with an additional supply of ChAdOx1 nCoV-19 (AstraZeneca, viral-vector) through COVAX. During the expansion phase (Q2–Q3 2021), Ad26.COV2.S (Janssen, single-dose viral-vector) and mRNA-1273 (Moderna) broadened population coverage. From 2022 onward, booster campaigns increasingly relied on monovalent mRNA platforms, initially targeting high-risk populations (≥70 years, transplant recipients, and immunocompromised individuals) before expanding to the general population.

The genomes from our cohort were distributed across multiple Omicron sublineages rather than clustering within a single lineage, consistent with repeated introductions and sustained community transmission during successive Omicron waves. Rapid lineage turnover occurred across 2022, with early circulation of 21K variants followed by mid-year emergence of 22A, 22B, and 22C lineages and later dominance of 22E. The mutational heatmap highlights the high number of mutations in Omicron genomes, particularly in Spike and ORF1a/ORF1b. When interpreted alongside Colombia’s vaccination timeline (Figure 7B), these observations indicate that many infections occurred during the transition from primary vaccination rollout to booster expansion, when booster coverage remained incomplete and intervals since the last dose were often prolonged.

## 4. Discussion

The study period (29 October 2021–7 February 2023) overlapped with Colombia’s initial booster vaccination campaign and followed three major SARS-CoV-2 waves (August 2020, January 2021, and June 2021). Our cohort largely represented a high-risk population, with 84% of participants meeting CDC criteria for increased risk of severe COVID-19. At enrollment, 54.9% of individuals showed evidence of prior infection based on nucleocapsid antibodies, 84.8% had received at least one vaccine dose, and 46% had received a booster dose. These findings reflect the epidemiologic context of the late pandemic, when population immunity was increasingly shaped by combined exposure to infection and vaccination.

In Colombia, the vaccination rollout started later than in high-income or vaccine-producing countries and was influenced by fluctuating vaccine availability, prioritization of high-risk populations, and unequal uptake. Early deployment relied largely on BNT162b2 and CoronaVac, followed by broader incorporation of viral vector and additional mRNA vaccines, while booster rollout progressed more gradually. This context likely explains the predominance of non-mRNA platforms in our cohort, the long interval since the last vaccine dose in many participants, and the incomplete booster coverage observed [6,33].

All patients in this study required hospitalization due to acute SARS-CoV-2 infection. Most infections occurred despite vaccination, including cases after booster administration. Baseline seropositivity was therefore high (81 to 85% across ELISA, CMIA, and neutralization assays). This contrasts with earlier seroprevalence estimates in Colombia during 2020, when population IgG seropositivity was substantially lower, ranging from 26% to 68% (approximately 26% in Medellín) [34]. Importantly, nucleocapsid antibody positivity was frequent not only among unvaccinated individuals, 65% of whom were seropositive, but also among vaccinated participants, indicating substantial prior natural exposure in both groups. Taken together, these findings reveal considerable heterogeneity in prior exposure history and support the interpretation that the immune profiles observed in this cohort reflect combined effects of vaccination and natural infection rather than isolated vaccine responses. Finally, the stronger neutralizing responses observed against Wuhan and Mu, compared with BA.1 and BA.2, support the view that the immune landscape in this cohort was shaped by cumulative, sequential, and overlapping exposures to vaccination and successive circulating variants over time, rather than by a single immunologic event. This interpretation is also consistent with reports showing reduced neutralization of early Omicron subvariants relative to ancestral strains after vaccination with platforms such as BNT162b2 and CoronaVac [35].

Patients ≥65 years showed higher antibody positivity rates, likely reflecting early vaccination prioritization. Nearly half of the cohort developed critical illness, with organ dysfunction and mortality concentrated among individuals with underlying conditions. The higher antibody positivity observed in patients aged ≥65 years, despite increased disease severity, likely reflects quantitative seropositivity rather than qualitative antibody functionality. In older individuals, immune responses are influenced by immunosenescence, which can result in preserved or even elevated antibody titers but with reduced neutralizing capacity, affinity, or coordination with cellular immunity. Additionally, more severe disease has been associated with stronger humoral responses, which may further contribute to higher detectable antibody levels in this group. These findings reflect global T-cell dynamics and that further studies incorporating antigen-specific and phenotypic markers would be required to definitively characterize memory formation [36,37,38].

Antibody responses persisted for 4 to 5 months after infection, consistent with evidence that complete vaccination, particularly with mRNA platforms, induces more durable immunity than infection alone or incomplete vaccination [39]. However, despite estimates of >88% protection against severe disease for up to 40 weeks after vaccination [40], and despite the fact that reinfection rarely progresses to critical illness [41], severe outcomes still occurred in our cohort, particularly among patients with underlying clinical risk factors. Our findings indicate that prior immunologic priming does not fully prevent severe disease in all individuals. In this setting, severe outcomes were likely driven by the combined effects of later Omicron sublineages, heterogeneous vaccine exposure, waning time since the last dose, and host vulnerability. Overall, these results underscore that the clinical impact of SARS-CoV-2 is shaped by the interaction between prior immunity, patient risk profile, and viral evolution [17,18,19,42,43,44,45,46].

Our analysis of cellular immunity supports a role for vaccination and booster doses in shaping the durability of T-cell responses over time. In particular, mRNA-based regimens appeared to confer a more sustained cellular response. This is consistent with previous evidence indicating that booster vaccination strengthens cellular immunity, with fully vaccinated individuals who receive an additional dose showing more robust CD4^+^ and CD8^+^ T-cell responses, particularly against Omicron, together with preserved cross-reactivity across Omicron, Delta, and ancestral strains [47,48].

In our cohort, the increase in CD4^+^ and CD8^+^ T-cell counts observed at 4 to 5 months is most consistent with a combination of recovery from acute lymphopenia and the establishment of post-infection immune homeostasis [49]. During acute COVID-19, lymphopenia is a well-described phenomenon, followed by gradual reconstitution of circulating T-cell populations during recovery [50]. Although most patients normalize lymphocyte counts within 6–8 weeks after symptom onset, a subset of moderate-to-severe survivors (7–12%) display persistent lymphopenia up to 17–50 days, suggesting that greater disease severity can imprint lasting immune dysregulation [51,52].

Our cohort mirrored the broader vaccination context in Colombia, which was characterized by multiple biologic platforms, variable schedules, delays in dose administration, and unequal vaccine availability. This may partly account for the limited use of heterologous vaccination in our cohort, despite evidence that such regimens, particularly when combined with mRNA boosters, may induce more robust humoral and cellular immunity than homologous schedules [53,54,55,56,57,58]. Recent studies indicate that heterologous vaccination schedules elicit higher CD4^+^ and CD8^+^ T-cell frequencies, greater anti-Spike IgG responses one year after vaccination, and superior neutralization of Omicron B.1.1.529/BA.2 relative to homologous regimens [54]. In addition, heterologous schedules may provide better protection against breakthrough infection in immunocompromised populations [59].

Omicron infection was associated with sustained reconstitution of T-cell responses, although this process appeared to be delayed in critically ill patients [60]. As neutralizing antibodies wane, cellular immunity likely assumes a more prominent role in maintaining durable protection [61,62]. This is especially relevant in the context of hybrid immunity, as seen in our cohort, where prior vaccination combined with natural infection may contribute to enhanced immune protection [63,64,65,66].

Several factors likely contributed to severe disease despite vaccination in our cohort. These include differences in vaccine platform, given the higher effectiveness reported for mRNA vaccines relative to some other platforms [66,67], despite the fact that only 39.6% of our participants received mRNA-based vaccines. Booster exposure may also have been relevant, as booster doses have been shown to significantly reduce breakthrough infections, although residual risk remains higher among individuals with comorbidities [65]. In parallel, viral evolution during the study period may have promoted immune escape [68], while immunosenescence and chronic comorbidities likely further compromised vaccine-induced protection in older adults [69,70].

Genomic analyses provide important context for these findings. The time-resolved phylogeny showed that genomes from our cohort were distributed across multiple Omicron sublineages rather than forming a single cluster, supporting repeated introductions and sustained community transmission during successive Omicron waves. In parallel, the mutational heatmap highlighted the substantial mutational burden of Omicron genomes, especially in the Spike protein and ORF1a/ORF1b regions [71]. Considered alongside Colombia’s vaccination timeline, these data indicate that many infections occurred under conditions of incomplete booster coverage, heterogeneous vaccine exposure, and prolonged intervals since the last dose. Together with the high mutational burden of Omicron, particularly in Spike, these factors likely favored continued transmission and viral persistence in the setting of partial immune escape [22,33].

Although Omicron was the predominant variant during the study period, these findings should be interpreted within the broader epidemiologic context of Colombia. Sustained co-circulation of multiple SARS-CoV-2 lineages, including the regionally significant Mu variant, together with heterogeneous vaccine exposure and variable timing of infection and vaccination, created a real-world model of complex hybrid immunity. In this sense, the study reflects the epidemiologic conditions of the late pandemic, when population immunity was increasingly shaped by the combined effects of natural infection and vaccination [20,21,22].

Overall, our findings highlight both the achievements and the limitations of current vaccination strategies. Vaccination, especially when combined with booster doses, enhances humoral and cellular immunity and significantly reduces the risk of severe disease and death [72,73]. However, severe COVID-19 continues to occur in vulnerable populations, emphasizing the need for adaptive vaccination strategies, including timely booster administration, variant-adapted vaccines, and targeted protection for high-risk individuals. Continued immunological and genomic surveillance will remain essential to guide vaccination policies and reduce the burden of COVID-19 among populations most susceptible to severe disease.

This study has important strengths, particularly the characterization of a hospitalized cohort with clinically relevant risk factors that reflects real-world patient populations. Importantly, it integrates longitudinal humoral and cellular immune profiling with detailed clinical phenotyping in a high-risk hospitalized cohort, providing insight into the interplay among immune responses, disease severity, and host factors. The findings highlight both the benefits and the limitations of current vaccination strategies in the context of ongoing viral evolution and population heterogeneity. The study is also valuable in that it reflects the structural inequities faced by many low- and middle-income countries during the pandemic, including limited access to advanced immunologic tools, vaccines, reagents, and other research resources. Within this context, our work contributes real-world immunologic data from a population that remains underrepresented in the global literature. However, several limitations should be acknowledged. The sample size was modest and was primarily calculated for mortality analysis. Technical constraints related to flow cytometry may also have influenced the precision of CD4^+^ and CD8^+^ T-cell quantification, and some vaccinated groups included relatively few participants, limiting the robustness of descriptive comparisons. Although DAPI was used during acquisition as a viability marker, it was not included in the final gating strategy because some samples showed low viability and limited cellular recovery, and repeat sampling was not feasible under the logistical and operational constraints of the pandemic period. Finally, although virus-specific cellular immune assays would have provided greater immunologic resolution than total T-cell counts, these approaches were not feasible within the logistical and resource limitations of the study setting.

## 5. Conclusions

This study offers a real-world contribution to the understanding of SARS-CoV-2 immunity in a complex and underrepresented setting. The coexistence of multiple viral lineages, including Mu, together with the use of different vaccine platforms, created a heterogeneous immune environment that allowed characterization of humoral and cellular responses under conditions of repeated antigenic stimulation and diverse vaccination histories. Our findings show that vaccination, particularly booster vaccination, enhanced immune responses over time; however, severe breakthrough infections still occurred, especially among high-risk individuals. Taken together, these results underscore both the benefits and the limitations of existing vaccination strategies and provide useful evidence for refining booster policies and strengthening preparedness for future respiratory viral threats in low- and middle-income countries.

## Data Availability

The raw data supporting the conclusions of this article will be made available by the authors on request.

## Supplementary Materials

The following supporting information can be downloaded at: https://www.mdpi.com/article/10.3390/vaccines14050394/s1, Table S1: Sequence of primers and probes in the Diagnose of Acute SARS-CoV.2; Table S2: Panel Of Immune Cells and Their Markers for Characterization; Figure S1: Gating Strategy Scheme; Table S3: Estimated sample size for various assumptions; Table S4: Characteristics of the Cohort with Acute SARS-CoV-2 Infection; Figure S2: Variant-specific analysis of neutralizing antibody (Wuhan, Mu, BA.1, BA.2). Additional Data, Methodological Details, and Results Are Presented in the Supplementary S1.
