# Supplementary material for: Hybrid and Vaccine-Induced Immunity Against SARS-CoV-2 in a Cohort of Hospitalized Patients from the Metropolitan Aburrá Valley, Colombia
Source: Vaccines (Basel). 2026 Apr 28;14(5):394. doi: 10.3390/vaccines14050394 (PMC13211762; doi:10.3390/vaccines14050394)
Supplement: Supplementary file 1 [file vaccines-14-00394-s001.zip › vaccines-4237649-supplementary.pdf]

Supplementary S1. ONLINE SUPPLEMENTARY MATERIAL

Hybrid and vaccine-induced immunity against SARS-CoV-2 in a cohort of hospitalized patients from the Metropolitan Aburrá Valley, Colombia

List of collaborators

Olga H. Hernández-Ortiz.MD, PhD. One Health Genomic Laboratory, Universidad Nacional de Colombia, Medellín, Colombia. Clínica Medellín- grupo Quirónsalud. Universidad de Antioquia, Medellín, Colombia. ORCID number: https://orcid.org/0000-0002-9738-0489
Email: ohelena.hernandez@udea.edu.co y olgahdezo@gmail.com

Andrés Felipe Naranjo. MD. Clínica Medellín- grupo Quirónsalud. Medellín, Colombia. ORCID number: https://orcid.org/0009-0003-5725-5813. Email: anaranjouci@gmail.com

Juan José Vélez Cadavid. MD. Hospital General de Medellín y Clínica las Américas. Medellín, Colombia. ORCID number: https://orcid.org/0009-0008-0246-5393Email: jvelez@hgm.gov.co

Bladimir Alejandro Gil Valencia. MD. MsC. Clínica las Américas-Auna. Medellín, Colombia. ORCID number: https://orcid.org/0000-0003-3521-6197 Email: bladigil@yahoo.com

Gisela de la Rosa Echavez. MD. Hospital Pablo Tobón Uribe. Medellín, Colombia. ORCID number: https://orcid.org/0000-0002-7690-0959. Email: giseladlr@gmail.com

A. Melissa Moreno. One Health Genomic Laboratory, Universidad Nacional de Colombia, Medellín, Colombia. ORCID number: https://orcid.org/0000-0002-5125-3011 Email: anrobledom@unal.edu.co

Laura S. Perez-Restrepo. One Health Genomic Laboratory, Universidad Nacional de Colombia, Medellín, Colombia. ORCID number: https://orcid.org/0000-0002-1734-3697 Email: lab_cwohc@unal.edu.co

Jaime Usuga. One Health Genomic Laboratory, Universidad Nacional de Colombia, Medellín, Colombia. ORCID number: https://orcid.org/0009-0005-2830-908X Email: jausugar@unal.edu.co

Manuela Aristizábal-Valencia. One Health Genomic Laboratory, Universidad Nacional de Colombia, Medellín, Colombia. ORCID: 0009-0009-3960-4024. Email: maaristizabal@unal.edu.co

Francisco Molina Saldarriaga. MD. MsC. PhD. Clínica Universitaria Bolivariana. Medellín, Colombia. ORCID number https://orcid.org/0000-0003-0705-6579 Email: francisco.molina@upb.edu.co

Jorge E. Sará Ochoa. MD. MsC. Orlando Family Physicians, Miami, Florida. ORCID number: https://orcid.org/0000-0002-7332-7456. Email: jeso72@gmail.com

Natalia Betancurt-Rodriguez. Department of Pathobiological Sciences, School of Veterinary Medicine, University of Wisconsin-Madison, Madison, Wisconsin. ORCID number:

Fabian Alberto Jaimes Barragán. MD. Esp. MSc. PhD. FACP. Universidad de Antioquia, Medellín, Colombia. https://orcid.org/0000-0002-7315-5367. Email: fabian.jaimes@udea.edu.co

Jorge E. Osorio. PhD. Department of Pathobiological Sciences, School of Veterinary Medicine, University of Wisconsin-Madison, Madison, Wisconsin. ORCID number: https://orcid.org/0000-0002-7474-6150.
Email: jorge.osorio@wisc.edu

Juan Pablo Hernández-Ortiz. PhD. One Health Colombia and One Health Genomic Laboratory, Universidad Nacional de Colombia, Medellín, Colombia. Departamento de Materiales y Nanotecnologia, Universidad Nacional de Colombia, Medellín, Colombia. ORCID number: https://orcid.org/0000-0003-0404-9947
Email: jphernandezo@wisc.edu, jphernandezo@unal.edu.co

1. **Methods**

Study Design and Settings

Definitions:

Acute SARS-CoV-2 infection: laboratory-confirmed diagnosis of acute SARS-CoV-2 infection (PCR for SARS-CoV-2, or Antigen) who presented with respiratory symptoms requiring hospitalization.

2. **Samples:**

Measurement of immune response to vaccination: We processed the samples as explained.

- **Diagnose of Acute SARS-CoV-2:** RNA extraction was performed on swab samples and nasopharyngeal washings using the ZR viral kit (Zymo® research, Irvine, CA, USA) following manufacturer´s instructions. rRT-PCR was performed in a thermocycler CFX96 (Bio-Rad Laboratories, Inc, USA) using iTaq Universal Probes One-Step Kit (Biorad®) and specific primers/probe targeting Envelope and RNA-dependent RNA polymerase (RdRP) genes3 (Table). Running conditions were: one cycle at 50°C for 10 min, one cycle at 95°C for 3 min and 40 cycles of 95°C for 15 s and 58°C for 30s. In addition, for each sample, an amplification of the Ribonuclease P gene (RNaseaP) was made as a control for the extraction of viral RNA. A Ct value was obtained from each of the samples according to the rRT-PCR curves; this value represents the cycle in which the samples have a positive amplification and exceed an RFU of 300 for the E and RdRP genes and of 100 for the Ribonuclease gene P. The samples were considered positive when the Ct value was less than 38. (See table S1). [28].

Table S1. Sequence of primers and probes in the Diagnose of Acute SARS-CoV-2

**Primers**	**Sequence of primers and probes**
E Sarbeco Forward	ACAGGTACGTTAATAGTTAATAGCGT
E Sarbeco Reverse	ATATTGCAGCAGTACGCACACA
E Sarbeco Probe	**FAM**-ACACTAGCCATCCTTACTGCGCTTCG-**BHQ-1**
RNasaP Forward	AGATTTGGACCTGCGAGCG
RNasaP Reverse	GAGCGGCTGTCTCCACAAGT
RNasaP Probe	**Cy5**-TTCTGACCTGAAGGCTCTGCGCG-**BHQ-1**
RdRP Forward	GTGARATGGTCATGTGTGGCGG
RdRP Reverse	CARATGTTAAASACACTATTAGCATA
RdRP Probe P1	**FAM**-CCAGGTGGWACRTCATCMGGTGATGC-**BBQ**
RdRP Probe P2	**FAM-**CAGGTGGAACCTCATCAGGAGATGC**-BBQ**

FAM: 6-carboxyfluorescein; BBQ: blackberry quencher; BHQ: black hole quencher

- **Whole genome sequencing**. Samples having a Ct below 27 were selected for sequencing using the nCoV-2019 sequencing protocol v3. cDNA was first made from the RNA (8 µl) and the LunaScript RT Supermix (5X) was used. Then, the multiplex PCR was performed using 218 primers, separated into 2 pools (pool 1 with 110 primers and pool 2 with 108 primers), and the Q5 hot start high fidelity 2X master mix was used (New England Biolabs, CITY, STATE). Two amplifications were conducted for each sample (3 µl of cDNA) and subsequently pooled by mixing 5 ul of each PCR product and 40 ul of nuclease-free water. Each amplicon pool was quantified with the aid of the Qubit ™ dsDNA HS Assay Kit (Thermo Fisher Scientific). The amplicon pools that had a concentration greater than or equal to 7ng / ul were used for the end repair. This was conducted using between 50 and 100 ng of DNA and the NEBNext® Ultra ™ II End Repair / dA-Tailing Module (New England Biolabs). Then the barcode ligation was made using the Native Barcoding Expansion 96 kit (Oxford Nanopore) and the Blunt / TA Ligase Master Mix (New England Biolabs). Each sample was pooled

and purified using AMPure XP beads (Beckman coulter). Next, the AMII was ligated using the Quick Ligation ™ Kit (New England Biolabs). DNA purification was performed again with the AMPure XP beads (Beckman coulter). Finally, the purified DNA was mixed with SQB buffer and Loading Beads (LB) and loaded into the Flow Cell (R9).
The assembly of raw NGS data was performed by following the pipeline described for Oxford Nanopore Technologies (ONT) platform. Each sequence obtained was analyzed with the help of Pango-Lineage, which assigns a lineage according to the lineages reported so far. In addition, Nextclade was used for the analysis of each of the mutations of the sequences.

- **Phylogenetic analysis**: The sequences from this study with references downloaded along with its metadata from Nextclade. They were aligned using Nextalign v1.2.1 as part of the Nextclade workflow (https://github.com/nextstrain/nextclade), using Wuhan-Hu-1 (NCBI Reference Sequence: NC045512.2) as reference. A Maximum likelihood Estimation (MLE) was inferred using IQ-Tree v2.1.29 with GTR+F+R2 substitution model estimated by means of the Bayesian Information Criterion (BIC) with the ModelFinder function10. Ultrafast bootstrap (UFboot)12 with 1000 replicates each to assess the tree topology and used iTOL v513 to visualize and analyze the resulting phylogenetic tree.

- **Qualitative detection of anti-SARS-CoV-2 antibodies**: Total IgG anti-Spike-SCoV-2 antibodies were determined using the SCoV-2 Detect IgG ELISA kit (Inbios International, Seattle, WA, USA) via qualitative indirect ELISA, according to the manufacturer's instructions. Optical densities (OD) were measured at 450 nm with a spectrophotometer (Multiskan Go, type 1510, Thermo Scientific). Interpretation of results was based on the immunological status ratio (ISR), with ISR <0.9 indicating a negative result and ISR >1.1 indicating a positive result for the presence of antibodies.

- **Quantitative detection of anti-SARS-CoV-2 antibodies:** Total IgG antibodies against the receptor-binding domain (RBD) of the SARS-CoV-2 spike protein were quantified using a chemiluminescent microparticle immunoassay (CMIA; ARCHITECT i1000 platform) with the Abbott SARS-CoV-2 IgG II Quant kit (Abbott, Chicago, IL, USA), according to the manufacturer's instructions. Antibody concentrations were analyzed using the instrument software and expressed in picogram units, in accordance with the assay's standardization. Results were interpreted according to the manufacturer's cut-offs: IgM negative if COV < 1.0 and reactive if ≥ 1.0; IgG negative if < 50.0 AU/mL and reactive if ≥ 50.0 AU/mL

- **Quantitative detection of anti-IgG nucleocapsid**: This assay was performed using Luminex xMAP technology with the anti-IgG nucleocapsid panel (Invitrogen Thermofisher), with results interpreted by the system software, and antibody titers reported in Median Fluorescence Intensity (MFI).
To evaluate the results, mean fluorescence intensity (MFI) values were used, incorporating controls from healthy individuals within each run to establish cutoff values between negative and positive PCR samples. The low control value was previously determined from a reference cohort consisting of 160 PCR-negative samples and 39 PCR-positive samples for SARS-CoV-2. Based on these values, a relative index was calculated using the following formula:

$$Ratio = \frac{MFI\ sample}{MFI\ low\ control}$$

The results were interpreted according to the following criteria:
  - Negative: ratio < 1.0: absence of specific immunoglobulins
  - Indeterminate: ratio between 1.0 and 1.3
  - Positive: ratio > 1.3: presence of specific immunoglobulins

Optionally, when a high-concentration control was available, standard curves were generated to allow for the interpolation of results and the calculation of relative quantitative values (U/mL), using specialized curve-fitting software in the ThermoFisher web page (https://identity.thermofisher.com/account-center/mfa-signin-identifier.html?gig_client_id=7n5vdUNnXIfn2Pt0x0HGAeDP)

- **Quantitative detection of anti-SARS-CoV-2 neutralizing antibodies**: The cPassTM SARS-CoV-2 Neutralization Antibody Detection Kit (GenScript) was utilized to detect neutralizing antibodies, which block the interaction between the virus and its receptor, against SARS-CoV-2, including virus variants such as Wuhan, Mu, BA.1, and BA.2, in serum samples, indicating potential prior infection or immune response.

  The **cPass™ SARS-CoV-2 Neutralization Antibody Detection Kit** is an ELISA-based assay designed to detect neutralizing antibodies against SARS-CoV-2 in human serum or plasma. This allows for the identification of individuals with an adaptive immune response to SARS-CoV-2, indicating prior infection. The kit works by simulating the interaction between the receptor-binding domain (RBD) of the SARS-CoV-2 spike protein and the angiotensin-converting enzyme 2 (ACE2) receptor, which is critical for viral entry into human cells. It detects neutralizing antibodies that block this interaction.

  Principle of the Assay:
  The assay begins with the RBD of the SARS-CoV-2 spike protein conjugated to horseradish peroxidase (HRP-RBD), which is incubated with the serum or plasma sample. If neutralizing antibodies are present, they bind to the RBD, preventing its interaction with ACE2. The mixture is then transferred to a microplate pre-coated with ACE2 protein, where only unbound HRP-RBD can attach to the ACE2. After washing away unbound components, a substrate is added, triggering a colorimetric reaction where HRP-RBD binds to ACE2. The color intensity, measured at a specific wavelength, is inversely proportional to the amount of neutralizing antibodies in the sample. The result is compared to a control to calculate the percentage of inhibition, providing a quantitative measure of neutralizing antibody levels.

  - Samples and supplied controls were diluted 1:10 with dilution buffer and mixed with HRP-RBD.
  - After incubation for 30 minutes at 37°C, 100 µl of samples, sample dilutions, or controls were added to a 96-well plate pre-coated with recombinant ACE2 protein.
  - The plate was incubated for 15 minutes at 37°C, followed by removal of the sample mixture and washing of the wells with the supplied washing buffer.
  - A substrate was added to initiate the reaction, which was stopped, and the plates were immediately read at 450 nm.
  - Data were interpreted as a percentage of reduction (% reduction) based on OD450 intensity. The manufacturer's recommended cutoff of ≥ 30% signal reduction was used to indicate the presence of SARS-CoV-2 neutralizing antibodies.

Flow Cytometry:
The antibodies used were based on the TBMNK Backbone Panel (Table S2).
Sample preparation was performed following the manufacturer's guidelines using TruCount™ tubes (Becton Dickinson, San Jose, CA, USA). The samples (50,000 events) were acquired using a flow cytometry analyzer (BD FACSLyric, BD Biosciences, San Jose, CA, USA) and analyzed with BD FACSuite v1.2.1 software. The median fluorescence intensity (MFI) of the markers in the acquired samples was further analyzed using FlowJo software (FlowJo, LLC, Portland, OR, USA).

The gating strategy included the exclusion of debris based on SSC versus FSC parameters, followed by the analysis of different cell populations. (Table S2 – Figure S1).

Table S2. Panels of Immune Cells and Their Markers for Characterization

Cell	Cell characterization	Marker
T Lymphocytes	Helper T Cells (CD4+)	CD3+ CD4+
	Cytotoxic T Cells (CD8+)	CD3+ CD8+

Figure S1. Gating Strategy Scheme

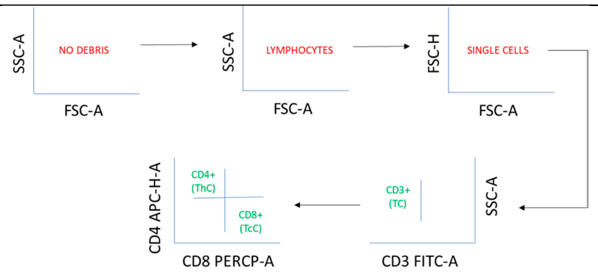

Figure S1: **Gating Strategy Scheme**
TC: T cells; ThC: T helper cells; TcC: cytotoxic T cell

<u>Sample size.</u> Bao et al.'s meta-analysis found that cellular immune response (CD4+ and CD8+ T cells) is halved in critically ill SARS-CoV-2 patients, while humoral response increases significantly, with IL-6 doubling and IL-10 increasing by 1.5 times compared to non-critical patients [a]. Critically ill patients have a 37% in-hospital mortality rate, compared to 11% in non-critical patients. About 32% of hospitalized patients require intensive care [b]. Assuming 30% mortality in critical patients (HR = 3) versus 10% in non-critical patients, with a critical-to-non-critical ratio of 1:4, alpha of 0.05, and power of 0.8, 82 patients are needed to detect mortality differences [c]. Adjustments were made for confounders, hypothetical SDs, and covariate correlations (R^2) following Hsieh and Lavori [d]. Therefore, the estimated sample sizes for various assumptions of the mentioned variables of interest (HR, SD, and R^2) were as follows: see Table S3

Table S3: Estimated sample sizes for various assumptions

Alpha	powder	Delta (Hazard Ratio)	Standar deviation (SD)	R^2	Probability of Death	Sample size (N) / Events (E)
0.05	0.8	3	0.5	0.1	0.2	N= 145 / E= 29
0.05	0.8	3	0.5	0.15	0.2	N= 154 / E= 31
0.05	0.8	3	0.55	0.1	0.2	N= 120 / E= 24
0.05	0.8	3	0.6	0.1	0.2	N= 101/ E= 21
0.05	0.8	3	0.45	0.1	0.2	N= 179 / E= 36
0.05	0.8	3	0.5	0.2	0.2	N= 163 / E= 33

Missing values

Missing data included one nucleocapsid antibody measurement, two CMIA spike antibody measurements, and ten neutralizing antibody measurements, representing 2.48% of all observations for these variables. Missing values were imputed using predictive mean matching with the mice package in R. This approach, based on Multivariate Imputation by Chained Equations, iteratively

imputes incomplete variables using models conditioned on the remaining observed data. The imputation model included sex, age, disease severity classification (moderate, severe, or critical), hypertension, diabetes, obesity, respiratory comorbidity, neoplasia, coronary artery disease, neurological disease, renal disease, vaccination status, and booster vaccination status.
The figure below shows the variable before and after imputation, with the red line indicating the original non-imputed values and the blue line indicating the imputed values. A corresponding table presenting the summary statistics for the original and imputed data is also included.

CMIA_ARCHITECT IgG: Missing: NA´s = 2

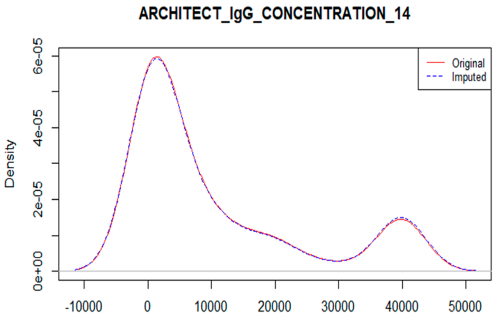

	ARCHITECT IgG CONCENTRATION	
	Original	Imputada
Min	0	0
1st cuartil	125.5	118.9
Mediana	3181.4	3181.4
Media	10231.7	10380.9
3rd Cuartil	15070.3	15226.4
Máximo	40000	40000

IgG ANTI NUCLEOCAPSID**:** Missing NA´s = 1

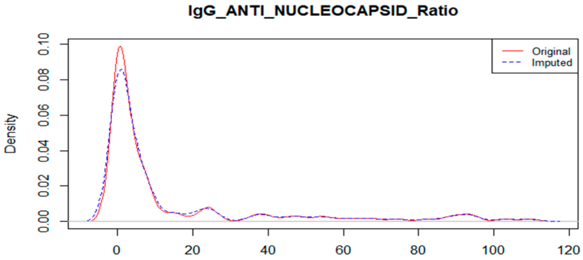

	IgG ANTI NUCLEOCAPSID Ratio	
	Original	Imputada
Min	0	0
1st cuartil	0.4563	0.4603
Mediana	19.603	19.643
Media	143.146	143.597
3rd Cuartil	91.855	108.583
Máximo	109.37	109.37

Wuhan**:** missing: NA´s = 10

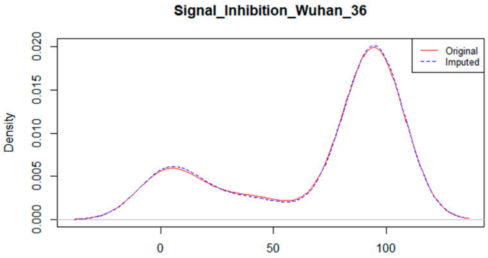

	Signal Inhibition Wuhan	
	Original	Imputada
Min	0	0
1st cuartil	39.59	37.94
Mediana	94.11	94.11
Media	70.04	69.95
3rd Cuartil	97.1	97.06
Máximo	97.61	97.61

Mu**:** Missing: NA´s = 10

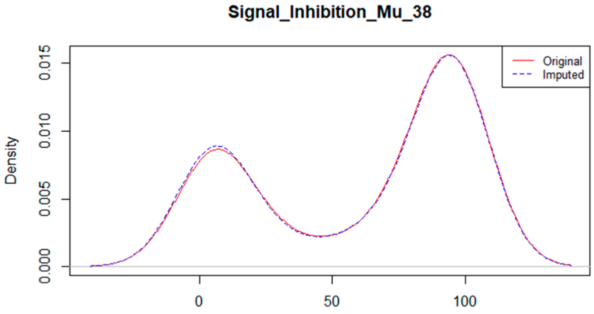

	Signal Inhibition Mu	
	Original	Imputada
Min	0	0
1st cuartil	10.13	9.8
Mediana	85.45	84.58
Media	61.77	61.21
3rd Cuartil	96.88	96.9
Máximo	98.2	98.2

BA.1: missing: NA´s = 10

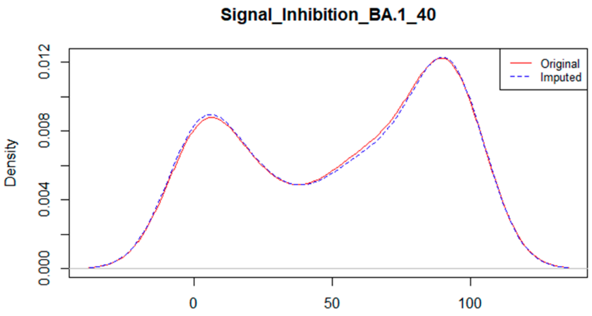

	Signal Inhibition BA.1	
	Original	Imputada
Min	0	0
1st cuartil	14.29	12.74
Mediana	60.71	60.32
Media	53.81	53.36
3rd Cuartil	90.62	90.62
Máximo	97.21	97.21

BA.2: missing: NA´s = 10

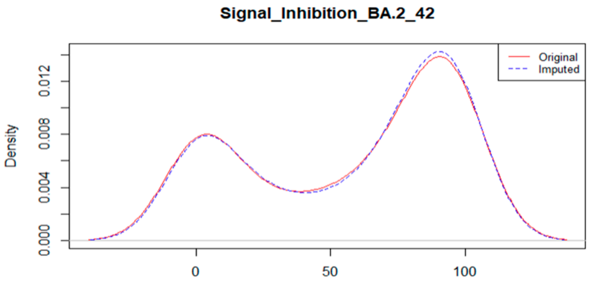

	Signal Inhibition BA.2	
	Original	Imputada
Min	0	0
1st cuartil	12.36	16.97
Mediana	76.29	76.98
Media	58	58.59
3rd Cuartil	94.84	94.91
Máximo	97.78	97.78

Statistical Methods

Serostatus dynamics using nucleocapsid IgG, ELISA IgG, and CMIA assays.

- Longitudinal changes in seropositivity between Measurement 1 and Measurement 4 were assessed using McNemar's test among participants with valid paired samples.

Contingency Table for NUCLEOCAPSID (Visit 1 vs Visit 4):

	Visit 4 Neg (0)	Visit 4 Pos (1)
Visit 1 Neg (0)	15	28
Visit 1 Pos (1)	6	44

McNemar's Test p-value: 3.1642e-04
Test used: Chi-Square with Yates correction
Conclusion: The change in the proportion of NUCLEOCAPSID positive cases between Visit 1 and Visit 4 is STATISTICALLY SIGNIFICANT ($p < 0.05$).

Contingency Table for CMIIA (Visit 1 vs Visit 4):

	Visit 4 Neg (0)	Visit 4 Pos (1)
Visit 1 Neg (0):	4	12
Visit 1 Pos (1):	1	76

McNemar's Test p-value: 3.4180e-03
Test used: Exact Binomial
Conclusion: The change in the proportion of positive cases is STATISTICALLY SIGNIFICANT ($p < 0.05$).

Contingency Table for ELISA (Visit 1 vs Visit 4):

	Visit 4 Neg (0)	Visit 4 Pos (1)
Visit 1 Neg (0)	1	12
Visit 1 Pos (1)	1	79

McNemar's Test p-value: 3.4180e-03
Test used: Exact Binomial
Conclusion: The change in the proportion of ELISA positive cases between Visit 1 and Visit 4 is STATISTICALLY SIGNIFICANT (p < 0.05).

- **Anti-SARS-CoV-2 antibodies by vaccination status and COVID-19 severity**

Cross-sectional analyses at Measurement 1 compared antibody positivity according to vaccination status and age group (<65 vs. ≥65 years), with additional stratification by disease severity (critical vs. non-critical); Fisher's exact test was used when subgroup sizes were small.

ANALYSIS FOR ELISA: CRITICAL PATIENTS

Contingency Table (Counts):

Vaccination_Status	Unvaccinated	Vaccinated
ELISA_Status		
Negative	5	5
Positive	8	30

Proportions of ELISA Positive:
- Unvaccinated: 8/13 (61.5%)
- Vaccinated: 30/35 (85.7%)

Statistical Results:
- Fisher's Exact P-value: 0.1075
- Odds Ratio: 3.7500. Conclusion: NOT statistically significant (p >= 0.05

ANALYSIS FOR ELISA: NON-CRITICAL PATIENTS

Contingency Table (Counts):

Vaccination_Status	Unvaccinated	Vaccinated
ELISA_Status		
Negative	3	7
Positive	4	69

Proportions of ELISA Positive:
- Unvaccinated: 4/7 (57.1%)
- Vaccinated: 69/76 (90.8%)

Statistical Results:
- Fisher's Exact P-value: 0.0348
- Odds Ratio: 7.3929. Conclusion: STATISTICALLY SIGNIFICANT (p < 0.05)

ANALYSIS FOR CMIA: CRITICAL PATIENTS (ARCHITECT):

Contingency Table (Counts):

Vaccination_Status	Unvaccinated	Vaccinated
Assay_Status		
Negative	7	5
Positive	6	30

Proportions of ARCHITECT Positive:
- Unvaccinated: 6/13 (46.2%)
- Vaccinated: 30/35 (85.7%)

Statistical Results:
- Fisher's Exact P-value: 0.0090
- Odds Ratio: 7.0000. Conclusion: STATISTICALLY SIGNIFICANT ($p < 0.05$)

ANALYSIS FOR CMIA: NON-CRITICAL PATIENTS (ARCHITECT)

Contingency Table (Counts):

Vaccination_Status	Unvaccinated	Vaccinated
Assay_Status		
Negative	3	10
Positive	4	66

Proportions of ARCHITECT Positive:
- Unvaccinated: 4/7 (57.1%)
- Vaccinated: 66/76 (86.8%)¡

Statistical Results:
- Fisher's Exact P-value: 0.0734
- Odds Ratio: 4.9500. Conclusion: NOT statistically significant ($p >= 0.05$)

ANALYSIS FOR NUCLEOCAPSID: CRITICAL PATIENTS

Contingency Table (Counts):

Vaccination_Status	Unvaccinated	Vaccinated
Assay_Status		
Negative	4	17
Positive	9	18

Proportions of NUCLEOCAPSID Positive:
- Unvaccinated: 9/13 (69.2%)
- Vaccinated: 18/35 (51.4%)

Statistical Results:
- Fisher's Exact P-value: 0.3377
- Odds Ratio: 0.4706 Conclusion: NOT statistically significant ($p >= 0.05$)

ANALYSIS FOR NUCLEOCAPSID: NON-CRITICAL PATIENTS

Contingency Table (Counts):

Vaccination_Status	Unvaccinated	Vaccinated
Assay_Status		
Negative	3	35
Positive	4	41

Proportions of NUCLEOCAPSID Positive:
- Unvaccinated: 4/7 (57.1%)
- Vaccinated: 41/76 (53.9%)

Statistical Results:
- Fisher's Exact P-value: 1.0000
- Odds Ratio: 0.8786 Conclusion: NOT statistically significant ($p >= 0.05$)

ANALYSIS FOR nABs: CRITICAL PATIENTS

Contingency Table (Counts):

Vaccination_Status	Unvaccinated	Vaccinated
Assay_Status		
Negative	8	7
Positive	5	28

Proportions of nABs Positive:
- Unvaccinated: 5/13 (38.5%)
- Vaccinated: 28/35 (80.0%)

Statistical Results:
- Fisher's Exact P-value: 0.0120
- Odds Ratio: 6.4000 Conclusion: STATISTICALLY SIGNIFICANT (p < 0.05)

ANALYSIS FOR nABs: NON-CRITICAL PATIENTS

Contingency Table (Counts):

Vaccination_Status	Unvaccinated	Vaccinated
Assay_Status		
Negative	4	13
Positive	3	63

Proportions of nABs Positive:
- Unvaccinated: 3/7 (42.9%)
- Vaccinated: 63/76 (82.9%)

Statistical Results:
- Fisher's Exact P-value: 0.0296
- Odds Ratio: 6.4615 Conclusion: STATISTICALLY SIGNIFICANT (p < 0.05)

ANALYZING ASSAY: ELISA - CRITICAL PATIENTS

Age_Group	< 65 Years	>= 65 Years
Assay_Status		
Negative	8	2
Positive	15	23

Proportions:
< 65 Years: 15/23 (65.2%)
>= 65 Years: 23/25 (92.0%)
Fisher's Exact P-value: 0.0335
Odds Ratio: 6.1333. Conclusion: SIGNIFICANT (p < 0.05)

ANALYZING ASSAY: ELISA - NON-CRITICAL PATIENTS

Age_Group	< 65 Years	>= 65 Years
Assay_Status		
Negative	5	5
Positive	35	38

Proportions:
< 65 Years: 35/40 (87.5%)
>= 65 Years: 38/43 (88.4%)
Fisher's Exact P-value: 1.0000
Odds Ratio: 1.0857. Conclusion: NOT SIGNIFICANT

ANALYZING ASSAY: CMIA (ARCHITECT) - CRITICAL PATIENTS

Age_Group	< 65 Years	>= 65 Years
Assay_Status		
Negative	9	3
Positive	14	22

Proportions:
< 65 Years: 14/23 (60.9%)
>= 65 Years: 22/25 (88.0%)
Fisher's Exact P-value: 0.0458
Odds Ratio: 4.7143. Conclusion: SIGNIFICANT ($p < 0.05$)

ANALYZING ASSAY: CMIA (ARCHITECT) - NON-CRITICAL PATIENTS

Age_Group	< 65 Years	>= 65 Years
Assay_Status		
Negative	9	4
Positive	31	39

Proportions:
< 65 Years: 31/40 (77.5%)
>= 65 Years: 39/43 (90.7%)
Fisher's Exact P-value: 0.1335
Odds Ratio: 2.8306. Conclusion: NOT SIGNIFICANT

ANALYZING ASSAY: NUCLEOCAPSID - CRITICAL PATIENTS

Age_Group	< 65 Years	>= 65 Years
Assay_Status		
Negative	8	13
Positive	15	12

Proportions:
- < 65 Years: 15/23 (65.2%)
- >= 65 Years: 12/25 (48.0%)

Fisher's Exact P-value: 0.2592
Odds Ratio: 0.4923. Conclusion: NOT SIGNIFICANT

ANALYZING ASSAY: NUCLEOCAPSID - NON-CRITICAL PATIENTS

Age_Group	< 65 Years	>= 65 Years
Assay_Status		
Negative	22	16
Positive	18	27

Proportions:
< 65 Years: 18/40 (45.0%)
>= 65 Years: 27/43 (62.8%)
Fisher's Exact P-value: 0.1258
Odds Ratio: 2.0625. Conclusion: NOT SIGNIFICANT

ANALYZING ASSAY: nABs - CRITICAL PATIENTS

Age_Group < 65 Years >= 65 Years

Assay_Status		
Negative	9	6
Positive	14	19

Proportions:
 < 65 Years: 14/23 (60.9%)
 >= 65 Years: 19/25 (76.0%)
Fisher's Exact P-value: 0.3532
Odds Ratio: 2.0357 Conclusion: NOT SIGNIFICANT

ANALYZING ASSAY: nABs - NON-CRITICAL PATIENTS

Age_Group	< 65 Years	>= 65 Years
Assay_Status		
Negative	7	10
Positive	33	33

Proportions:
 < 65 Years: 33/40 (82.5%)
 >= 65 Years: 33/43 (76.7%)
Fisher's Exact P-value: 0.5926
Odds Ratio: 0.7000 Conclusion: NOT SIGNIFICANT

- **Dynamics of CD4$^+$ and CD8$^+$ T-cell responses**

To assess longitudinal changes in CD4+ and CD8+ T-cell subsets across the four predefined time points, linear mixed-effects models were fitted to log10(x+1)-transformed absolute counts, with time point, primary vaccine platform, booster strategy, and their interactions included as fixed effects, and participant identifier included as a random effect.

Results for CD8:

MODEL 1: CD8+ DYNAMICS BY PRIMARY VACCINE (Fig 4A)
Mixed-Effects Model Convergence: True
Interaction P-values:

C(Time)[T.M2]:C(Primary_Vaccine)[T.mRNA]	0.197535
C(Time)[T.M3]:C(Primary_Vaccine)[T.mRNA]	0.189022
C(Time)[T.M4]:C(Primary_Vaccine)[T.mRNA]	0.045892
C(Time)[T.M2]:C(Primary_Vaccine)[T.Viral Vector]	0.333360
C(Time)[T.M3]:C(Primary_Vaccine)[T.Viral Vector]	0.743864
C(Time)[T.M4]:C(Primary_Vaccine)[T.Viral Vector]	0.367369
C(Time)[T.M2]:C(Primary_Vaccine)[T.Inactivated]	0.439923
C(Time)[T.M3]:C(Primary_Vaccine)[T.Inactivated]	0.841996
C(Time)[T.M4]:C(Primary_Vaccine)[T.Inactivated]	0.120552

MODEL 2: CD8+ DYNAMICS BY BOOSTER STRATEGY (Fig 4B)
Mixed-Effects Model Convergence: True
Interaction P-values

C(Time)[T.M2]:C(Booster_Strategy)[T.Homologous]	0.270445
C(Time)[T.M3]:C(Booster_Strategy)[T.Homologous]	0.493010
C(Time)[T.M4]:C(Booster_Strategy)[T.Homologous]	0.023950
C(Time)[T.M2]:C(Booster_Strategy)[T.Heterologous]	0.307042
C(Time)[T.M3]:C(Booster_Strategy)[T.Heterologous]	0.221802

C(Time)[T.M4]:C(Booster_Strategy)[T.Heterologous]   0.266001

POST-HOC T-TESTS FOR SPECIFIC MANUSCRIPT CLAIMS
Acute CD8+ Expansion (M1 vs M2)
[mRNA] M1 vs M2: Increased | p-value = 0.0850 (ns)
[Viral Vector] M1 vs M2: Increased | p-value = 0.2973 (ns)
[Inactivated] M1 vs M2: Increased | p-value = 0.3534 (ns)
[Unvaccinated] M1 vs M2: Increased | p-value = 0.9682 (ns)

Acute CD8+ Expansion (M1 vs M3)
[mRNA] M1 vs M3: Increased | p-value = 0.0200 **SIGNIFICANT**
[Viral Vector] M1 vs M3: Increased | p-value = 0.3636 (ns)
[Inactivated] M1 vs M3: Increased | p-value = 0.3682 (ns)
[Unvaccinated] M1 vs M3: Increased | p-value = 0.5419 (ns)

Long-term Immunity at 4-5 months (M4: Vaccinated vs Unvaccinated)
Vaccinated vs Unvaccinated at M4 | p-value = 0.1351 (ns)

Results for CD4:

MODEL 1: CD4+ DYNAMICS BY PRIMARY VACCINE
Mixed-Effects Model Convergence: True
Interaction P-values
C(Time)[T.M2]:C(Primary_Vaccine)[T.mRNA]   0.330306
C(Time)[T.M3]:C(Primary_Vaccine)[T.mRNA]   0.561821
C(Time)[T.M4]:C(Primary_Vaccine)[T.mRNA]   0.363736
C(Time)[T.M2]:C(Primary_Vaccine)[T.Viral Vector]   0.516745
C(Time)[T.M3]:C(Primary_Vaccine)[T.Viral Vector]   0.396240
C(Time)[T.M4]:C(Primary_Vaccine)[T.Viral Vector]   0.602919
C(Time)[T.M2]:C(Primary_Vaccine)[T.Inactivated]   0.951904
C(Time)[T.M3]:C(Primary_Vaccine)[T.Inactivated]   0.563053
C(Time)[T.M4]:C(Primary_Vaccine)[T.Inactivated]   0.314858

MODEL 2: CD4+ DYNAMICS BY BOOSTER STRATEGY
Mixed-Effects Model Convergence: True
Interaction P-values
C(Time)[T.M2]:C(Booster_Strategy)[T.Homologous]   0.441837
C(Time)[T.M3]:C(Booster_Strategy)[T.Homologous]   0.121743
C(Time)[T.M4]:C(Booster_Strategy)[T.Homologous]   0.059740
C(Time)[T.M2]:C(Booster_Strategy)[T.Heterologous]   0.872961
C(Time)[T.M3]:C(Booster_Strategy)[T.Heterologous]   0.238341
C(Time)[T.M4]:C(Booster_Strategy)[T.Heterologous]   0.456332

POST-HOC T-TESTS FOR SPECIFIC MANUSCRIPT CLAIMS
Acute CD4+ Change (M1 vs M2)
[mRNA] M1 vs M2: Increased | p-value = 0.0882 (ns)
[Viral Vector] M1 vs M2: Increased | p-value = 0.3071 (ns)
[Inactivated] M1 vs M2: Increased | p-value = 0.6892 (ns)
[Unvaccinated] M1 vs M2: Increased | p-value = 0.4735 (ns)
Acute CD4+ Change (M1 vs M3)
[mRNA] M1 vs M3: Increased | p-value = 0.0266 *SIGNIFICANT*

[Viral Vector] M1 vs M3: Increased | p-value = 0.5800 (ns)
[Inactivated] M1 vs M3: Increased | p-value = 0.4932 (ns)
[Unvaccinated] M1 vs M3: Increased | p-value = 0.1098 (ns)

Long-term Immunity at 4-5 months (M4: Vaccinated vs Unvaccinated)
Vaccinated vs Unvaccinated at M4 | p-value = 0.1032 (ns)

Results.

Table S4: Characteristics of the Cohort with Acute SARS-CoV-2 Infection

Variables	COVID-19 Total (N=131)	Critical Disease (N=48)	Non-critical Disease (N=83)
**Demographic characteristics**			
Age (years): Median (IQR)	65 (56–75.5)	65.0 (56.0–73.0)	65.0 (56.3–76.8)
Age ≥ 65 years, no (%)	68 (51.9%)	25 (52.0%)	43 (51.8%)
Male sex, no (%)	61 (46.6%)	23 (47.9%)	38 (45.8%)
**Comorbidities**			
Diabetes mellitus, no (%)	32 (24.4%)	12 (25.0%)	20 (24.1%)
Obesity, no (%)	52 (39.6%)	22 (45.8%)	30 (36.1%)
Hypertension, no (%)	72 (55.0%)	28 (58.3%)	44 (53.0%)
Chronic pulmonary disease, no (%)	32 (24.4%)	13 (27.1%)	19 (22.9%)
Neoplasms, no (%)	20 (15.2%)	6 (12.5%)	14 (16.9%)
Weakened immune system, no (%)	34 (26.0%)	11 (22.9%)	23 (27.7%)
High-risk condition, no (%)	106 (80.9%)	41 (85.4%)	65 (78.3%)
Medical condition, no (%)	110 (84.0%)	41 (85.4%)	69 (83.1%)
**COVID-19 symptoms**			
Cough, no (%)	96 (73.2)	38 (79.2)	58 (69.9)
Fever, no (%)	65 (49.6)	28 (58.3)	37 (44.6)
Dyspnea, no (%)	80 (61.1)	36 (75.0)	44 (53.0)
Taste/Smell alteration, no (%)	15 (11.5)	9 (18.8)	6 (7.2)
**Chest Tomographic involvement at admission**			
Ground-glass/ cobblestone pattern, no (%)	58 (44.3%)	35 (71.4%)	22 (28.0%)
Bilateral or diffuse infiltrates, no (%)	55 (42.0%)	35 (71.4%)	20 (24.4%)
**Status on day 28 of COVID-19 infection**			
28-day mortality, no (%)	10 (7.6%)	9 (18.8%)	1 (1.2%)

Chronic pulmonary disease (CPD): Includes asthma and chronic obstructive pulmonary disease (COPD). Neoplasia: Includes solid cancers, leukemia, or lymphoma.
Weakened immune system (WIS): Includes neoplasia, solid organ or hematopoietic stem cell transplantation, HIV infection, lupus, or rheumatoid arthritis.
High-risk condition (HR): Includes CPD, diabetes (type 1 or 2), obesity, hypertension, chronic kidney disease (CKD), cerebrovascular disease, or coronary artery disease.
Medical conditions: Comorbidities classified under WIS or HR

Variant-specific analysis of neutralizing antibody (Wuhan, Mu, BA.1, BA.2) revealed that, at baseline, positivity was highest for the Wuhan variant (n = 99; 75.6%) and BA.2 (n = 94; 71.8%). At 4-5 months, positivity rates were highest for BA.1 and BA.2 (**Figure S2**).

Figure S2. Variant-specific analysis of neutralizing antibody (Wuhan, Mu, BA.1, BA.2)

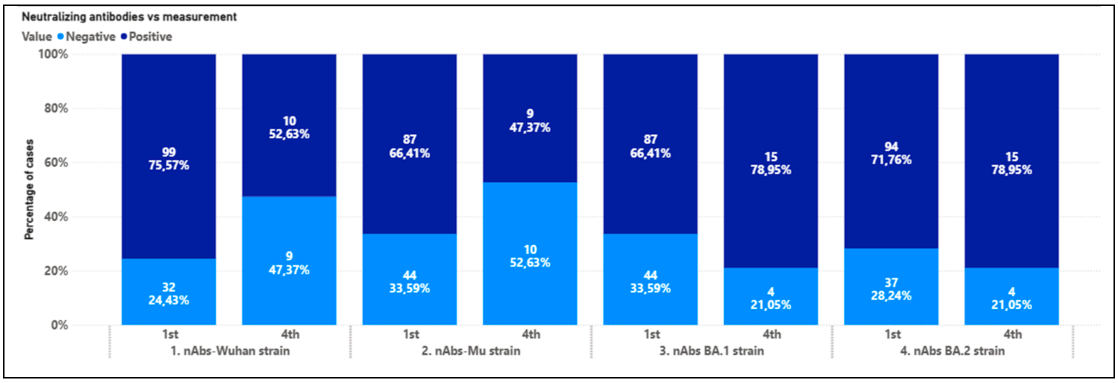

To adhere to the **STROBE (**Strengthening the Reporting of Observational Studies in Epidemiology **recommendations)**, we have included the required additional details:

Codification

Variables	Definition	Codification
Age	Age in years at ICU admission	
Sex	Gender of participant	1= males<br>0= female
**Income**	Categorized into two groups: low income and high income	<3 low income<br>≥4 high income
Diabetes	Diagnosed with Diabetes Type I or II	1= yes<br>0= no
Obesity	Having antecedent of obesity (BMI is 30 kg/m^2 or higher	1= yes<br>0= no
Hypertension	Diagnosed with hypertension	1= yes<br>0= no
**Asthma**	Diagnosed with asthma	1= yes<br>0= no
**Chronic obstructive pulmonary disease (COPD).**	Diagnosed with COPD	1= yes<br>0= no
Heart disease	Diagnosed with heart failure or coronary artery disease	1= yes<br>0= no
**Chronic kidney disease (CKD)**	Diagnosed with CKD	1= yes<br>0= no
**Neurological conditions**	Diagnosed with cerebrovascular disease	1= yes<br>0= no
**Chronic liver disease**	Diagnosed with chronic liver disease	1= yes<br>0= no
**Cancer**	Having antecedent or diagnosed with of solid cancer	1= yes<br>0= no
**leukemia, or lymphoma**	Having antecedent of diagnosed with leukemia, or lymphoma	1= yes<br>0= no
**HIV infection**	Diagnosed with of Human Immunodeficiency Virus	1= yes<br>0= no
**Chronic lung disease (CLD)**	Antecedent of asthma or chronic obstructive pulmonary disease (COPD).	1= yes<br>0= no
**Neoplasia**	Antecedent of solid cancers, leukemia, or lymphoma	1= yes<br>0= no
**Weakened immune system (WIS):**	Antecedent of Neoplasia and solid organ or blood stem cell transplant, HIV infection, Lupus, or rheumatoid arthritis.	1= yes<br>0= no
**High-risk (HR) condition:**	Antecedent of CLD, Diabetes (type 1 or 2), obesity, chronic kidney disease (CKD), tuberculosis, cerebrovascular disease, coronary disease, and/or hypertension.	1= yes<br>0= no
**Medical condition:**	Antecedent of WIS and/or HR condition.	1= yes<br>0= no
**Vaccination status**	Complete vaccination schedule (CVS) required:<br>• Second dose of CoronaVac, Moderna, and AstraZeneca administered 28 days after the first dose<br>• Second dose of Pfizer scheduled 21 days after the first dose.<br>• Single dose of Janssen	Complete: 1<br>Incomplete:0
**Days of vaccination scheduled**	Calculated by subtracting the date of second dose of vaccination from the baseline survey date.	Date: dd/mm/year
**Time of vaccination scheduled**	Calculated by dividing the number of vaccination days by 30, and then classifying it as either greater or less than four months.	1: ≥ 4 months<br>0: < 4 months
**Booster vaccinations**	Receive first-time booster vaccinations	1: yes<br>0: no
**Days of booster scheduled**	Calculated by subtracting the date of booster of vaccination from the baseline survey date.	Date: dd/mm/year
**Time of booster scheduled**	Calculated by dividing the number of booster days by 30, and then classifying it as either greater or less than four months.	1: ≥ 4 months<br>0: < 4 months

**Qualitative detection of anti SARS-CoV-2 antibodies**	SCoV-2 Detect ™ IgG ELISA is an in vitro diagnostic test for the qualitative detection of IgG antibodies against SARS-CoV-2 in serum performed manually.	1: positive<br>0: negative
**Quantitative and qualitative detection of anti SARS-CoV-2 antibodies**	IgG antibodies against SARS-CoV-2 were quantified using a chemiluminescent microparticle immunoassay (CMIA; ARCHITECT i System), with a cutoff of 50.0 AU/mL.	1: positive ≥ 50<br>0: negative < 50
**Qualitative detection of anti IgG nucleocapsid**	Luminex xMAP technology with the anti-IgG nucleocapsid panel (Invitrogen Thermofisher), with results interpreted by the system software, and antibody titers reported in Median Fluorescence Intensity (MFI).	1: positive<br>0: negative
**Quantitative detection of anti SARS-CoV-2 neutralizing antibodies**<br>**Wild/Wuhan Variant (Lote: A221101)**<br>**Mu Variant (Lote: A221102)**<br>**BA.1 Variant (Lote: A221103)**<br>**BA.2 Variant (Lote: A221104)**	cPass™ SARS-CoV-2 Neutralization Antibody Detection Kit, allows the qualitative and rapid detection of total neutralizing antibodies (NAbs) in human serum.	1: Positive ≥ 30%<br>0: Negative< 30%

Table: Reporting STROBE and RECORD checklists.

		STROBE items	RECORD items	Location in manuscript where items are reported
**Title and abstract**				
	1	(a) Indicate the study's design with a commonly used term in the title or the abstract (b) Provide in the abstract an informative and balanced summary of what was done and what was found	RECORD 1.1: The type of data used should be specified in the title or abstract. When possible, the name of the databases used should be included.<br>RECORD 1.2: If applicable, the geographic region and timeframe within which the study took place should be reported in the title or abstract.<br>RECORD 1.3: If linkage between databases was conducted for the study, this should be clearly stated in the title or abstract.	p. 1-2
**Introduction**				
Background rationale	2	Explain the scientific background and rationale for the investigation being reported		p. 2-3
Objectives	3	State specific objectives, including any prespecified hypotheses		p. 3
**Methods**				
Study Design	4	Present key elements of study design early in the paper		P p. 3-4
Setting	5	Describe the setting, locations, and relevant dates, including periods of recruitment, exposure, follow-up, and data collection		p. 3-4
Participants	6	*(a) Cohort study* - Give the eligibility criteria, and the sources and methods of selection of participants. Describe methods of follow-up<br>*Case-control study* - Give the eligibility criteria, and the sources and methods of case ascertainment and control selection. Give the rationale for the choice of cases and controls<br>*Cross-sectional study* - Give the eligibility criteria, and the sources and methods of selection of participants<br><br>*(b) Cohort study* - For matched studies, give matching criteria and number of exposed and unexposed<br>*Case-control study* - For matched studies, give matching criteria and the number of controls per case	RECORD 6.1: The methods of study population selection (such as codes or algorithms used to identify subjects) should be listed in detail. If this is not possible, an explanation should be provided.<br><br>RECORD 6.2: Any validation studies of the codes or algorithms used to select the population should be referenced. If validation was conducted for this study and not published elsewhere, detailed methods and results should be provided.<br><br>RECORD 6.3: If the study involved linkage of databases, consider use of a flow diagram or other graphical display to demonstrate the data linkage process, including the number of individuals with linked data at each stage.	p. 3-6

Variables	7	Clearly define all outcomes, exposures, predictors, potential confounders, and effect modifiers. Give diagnostic criteria, if applicable.	RECORD 7.1: A complete list of codes and algorithms used to classify exposures, outcomes, confounders, and effect modifiers should be provided. If these cannot be reported, an explanation should be provided.	p.4-5, appendix p.16-17
Data sources/ measurement	8	For each variable of interest, give sources of data and details of methods of assessment (measurement). Describe comparability of assessment methods if there is more than one group		p. 5-6
Bias	9	Describe any efforts to address potential sources of bias		p. 6
Study size	10	Explain how the study size was arrived at		Appendix p.5
Quantitative variables	11	Explain how quantitative variables were handled in the analyses. If applicable, describe which groupings were chosen, and why		p.6
Statistical methods	12	(a) Describe all statistical methods, including those used to control for confounding<br>(b) Describe any methods used to examine subgroups and interactions<br>(c) Explain how missing data were addressed<br>(d) *Cohort study* - If applicable, explain how loss to follow-up was addressed<br>*Case-control study* - If applicable, explain how matching of cases and controls was addressed<br>*Cross-sectional study* - If applicable, describe analytical methods taking account of sampling strategy<br>(e) Describe any sensitivity analyses		p. 7
Data access and cleaning methods		..	RECORD 12.1: Authors should describe the extent to which the investigators had access to the database population used to create the study population.<br><br>RECORD 12.2: Authors should provide information on the data cleaning methods used in the study.	NA
Linkage		..	RECORD 12.3: State whether the study included person-level, institutional-level, or other data linkage across two or more databases. The methods of linkage and methods of linkage quality evaluation should be provided.	NA
**Results**				

Participants	13	(a) Report the numbers of individuals at each stage of the study (*e.g.*, numbers potentially eligible, examined for eligibility, confirmed eligible, included in the study, completing follow-up, and analysed)<br>(b) Give reasons for non-participation at each stage.<br>(c) Consider use of a flow diagram	RECORD 13.1: Describe in detail the selection of the persons included in the study (*i.e.,* study population selection) including filtering based on data quality, data availability and linkage. The selection of included persons can be described in the text and/or by means of the study flow diagram.	p. 7-8
Descriptive data	14	(a) Give characteristics of study participants (*e.g.*, demographic, clinical, social) and information on exposures and potential confounders<br>(b) Indicate the number of participants with missing data for each variable of interest<br>(c) *Cohort study* - summarise follow-up time (*e.g.*, average and total amount)		p. 7-17
Outcome data	15	*Cohort study* - Report numbers of outcome events or summary measures over time<br>*Case-control study* - Report numbers in each exposure category, or summary measures of exposure<br>*Cross-sectional study* - Report numbers of outcome events or summary measures		p. 7-17
Main results	16	(a) Give unadjusted estimates and, if applicable, confounder-adjusted estimates and their precision (e.g., 95% confidence interval). Make clear which confounders were adjusted for and why they were included<br>(b) Report category boundaries when continuous variables were categorized<br>(c) If relevant, consider translating estimates of relative risk into absolute risk for a meaningful time period		p.7-11 and appendix p. 14-15
Other analyses	17	Report other analyses done—e.g., analyses of subgroups and interactions, and sensitivity analyses		Appendix 7-14
**Discussion**				
Key results	18	Summarise key results with reference to study objectives		p. 20
Limitations	19	Discuss limitations of the study, taking into account sources of potential bias or imprecision. Discuss both direction and magnitude of any potential bias	RECORD 19.1: Discuss the implications of using data that were not created or collected to answer the specific research question(s). Include discussion of misclassification bias, unmeasured confounding, missing data, and changing eligibility over time, as they pertain to the study being reported.	p. 2

Interpretation	20	Give a cautious overall interpretation of results considering objectives, limitations, multiplicity of analyses, results from similar studies, and other relevant evidence		p. 20-21
Generalisability	21	Discuss the generalisability (external validity) of the study results		p. 20-21
**Other Information**				
Funding	22	Give the source of funding and the role of the funders for the present study and, if applicable, for the original study on which the present article is based		p. 21
Accessibility of protocol, raw data, and programming code		..	RECORD 22.1: Authors should provide information on how to access any supplemental information such as the study protocol, raw data, or programming code.	NA

STROBE: Strengthening the Reporting of Observational studies in Epidemiology. RECORD: Reporting of Studies Conducted using Observational Routinely-collected Data.
NA: not applicable
